# Grain nutritional and antioxidant profiling of diverse lentil (*Lens culinaris* Medikus) genetic resources revealed genotypes with high nutritional value

**DOI:** 10.3389/fnut.2024.1344986

**Published:** 2024-03-22

**Authors:** Fareeha Riaz, Amjad Hameed, Muhammad Jawad Asghar

**Affiliations:** Nuclear Institute for Agriculture and Biology College, Pakistan Institute of Engineering and Applied Sciences (NIAB-C, PIEAS), Faisalabad, Pakistan

**Keywords:** lentil, biochemical profiling, nutritional traits, principal component analysis, cluster analysis, antioxidants, correlation

## Abstract

The lentil (*Lens culinaris* Medikus ssp. Culinaris) is a self-pollinating, diploid (2n = 2X = 14) crop with a genome size of 4 Gbp. The present study was conducted to provide a database for the evaluation of lentil antioxidant capacity, nutritional quality, and biochemical attributes. For these purposes, lentil germplasm, including 100 exotic and local genotypes from different agro-climatic zones of Pakistan, was collected. Significant variation (*p* < 0.05) was found among the genotypes under investigation using the Tukey HSD test. Ascorbate peroxidase was highest in ALTINOPARK (2,465 Units/g s. wt.), catalase in LPP 12110 (5,595 Units/g s. wt.), superoxide dismutase in LPP 12105 (296.75 Units/g s. wt.), and peroxidase in NIAB Masoor 2002 (3,170 Units/g s. wt.). Furthermore, NLM 15016 had a maximum total antioxidant capacity of 15.763 mg/g s. wt. The maximum values of total soluble sugars (83.93 mg/g. s. wt.) and non-reducing sugars (74.79 mg/g. s. wt.) were noticed in NLM 15015. The highest reducing sugars were detected in ILL 8006 (45.68 mg/g. s. wt.) ascorbic acid in LPP 12182 (706 μg/g s. wt.), total phenolic content in NLI 17003 (54,600 μM/g s. wt.), and tannins in NLI 17057 (24,563 μM/g s. wt.). The highest chlorophyll a (236.12 μg/g s. wt.), chlorophyll b (317 μg/g s. wt.), total chlorophyll (552.58 μg/g s. wt.), and lycopene (10.881 μg/g s. wt.) were found in NLH 12097. Maximum total carotenoids were revealed in the local approved variety Markaz 2009 (17.89 μg/g s. wt.). Principal component analysis (PCA), correlation analysis (Pearson’s test), and agglomerative hierarchical clustering (AHC) were performed to detect the extent of variation in genotypes. In cluster analysis, all genotypes were categorized into three clusters. Cluster II genotypes showed remarkable divergence with cluster III. According to PCA, the contribution of PC-I regarding tested nutritional parameters toward variability was the highest (39.75%) and indicated positive factor loading for the tested nutritional and biochemical parameters. In conclusion, genotype X 2011S 33–34-32 can be used by the food industry in making pasta, multigrain bread, and snacking foods due to its high protein content for meat alternative seekers. Identified genotypes with high nutritional attributes can be utilized to improve quality parameters in the respective lentil breeding lines.

## Introduction

In recent years, grain legumes have acquired remarkable importance in the food industry and among consumers. This consumption pattern is primarily accelerated by consumers based on environment-supportable plant-based proteins (1, 2). The increased legume utilization and consumption are attributed to education levels and knowledge among consumers that significantly enhance the issues regarding food nutritional profile, health benefits, and food quality (3). The health benefits of legumes are clearly associated with certain antioxidants present in them. These antioxidants retard the oxidation of several oxidizable molecules and, hence, play crucial roles in preventing excessive production of reactive oxygen species (ROS) or free radicals. Free radicals are unstable, harmful molecules, which are produced as a result of an imbalance between antioxidant enzymes and reactive oxygen species. Furthermore, antioxidants are the crucial constituents of various diets that scavenge ROS, or free radicals, and thus impede oxidative mechanisms (2). Synthetic antioxidants are synthesized commercially and are further utilized in various industrial approaches. On the other hand, natural antioxidants are mainly found in various fruits and legumes, such as chickpeas, lentils, peas, and beans (4, 5). These natural antioxidants are continuously gaining enormous importance among food industry nutritionists due to their therapeutic and safety potential (6). In order to maintain regular biological activities and functions, a suitable balance is necessary between free radicals and antioxidants. Enzymatic antioxidants, for example, ascorbate peroxidase (APX) and superoxide dismutase (SOD), have the ability to eliminate hydrogen peroxide and other reactive oxygen species (ROS) (7, 8). Non-enzymatic antioxidants consist of two classes: membrane-bound (lipid-soluble), such as beta-carotene and alpha-tocopherol, and water-soluble reducing agents like phenolic compounds and ascorbic acid (9).

Pulses, including lentil (*Lens culinaris Medikus*), have attained immense popularity among food processors and consumers in recent times. The increasing demand for lentils is due to users opting for environmentally sustainable food resources and plant-based proteins. The global lentil production has increased 2-fold since 2001 (from 3.15 to 6.58 million metric tons in 2020), which reveals the commercial and domestic importance of this nutrient-rich legume (1).

The utilization of legumes, such as lentils, is gaining escalating recognition in multiple food applications. Because of their balanced protein and fat content, lentil flour represents the best option for the formation of novel and high-performing grain flour products (10–12). Overall, lentil flour can replace wheat flour with improved nutritional profile (13), reduced total carbohydrate content, significantly elevated protein, flavonoids, and antioxidant potential (14). Among legumes, lentil has gained immense importance in quality and production worldwide. Lentils are sown in more than 40 countries, with Canada, Turkey, Australia, the United States, and India being the largest producers (15). In the early 1980s, lentil was introduced to North America as a new crop to the preexisting crop rotation. Currently, lentil is considered as a major legume crop in both the United States and Canada (16). It is mainly cultivated in Southeast Asia and consumed as a soup made from split pulse (dhal) or as a whole grain. It is used in many culinary dishes in the Middle East and Southeast Asia and incorporated in broths and soups in North America and European countries (17). Lentil is the second largest cultivated legume (Rabi season) crop in Pakistan after chickpea (*Cicer arietinum* L.), both in quantity and quality (18). It is grown to 5% area under pulse production (19).

In addition to their remarkable nutritional profile, lentils also exhibit a number of phytochemicals, e.g., flavonoids, tocopherols, and carotenoids, and are well known to have the highest antioxidant properties (20, 21) given the existence of several bound and extractable phenolic compounds (22). The two major phenolic compounds present in lentils are phenolic acids and condensed tannins (20). Additionally, lentils contain several groups of phenolic compounds, which act as antioxidants. These antioxidant groups have the ability to suppress the formation of many reactive oxygen species (ROS) (23, 24).

However, the new trends in pulse consumption and their possible health benefits demand further investigation of certain antioxidant agents present in legumes, like lentils (25). There is a dire need to further investigate the possible antioxidant compounds present in lentils (2). To date, inadequate information is available for further evaluation of the nutritional, antioxidant capacity, and biochemical profiles of several diverse lentil genotypes. Keeping this in mind, the current study aimed to unveil the nutritional and biochemical attributes of Pakistani and exotic lentil genotypes. These explored genotypes with diverse nutraceutical properties can be utilized by nutraceuticals and lentil-based food industries for the development of lentil-based food byproducts. In the future, prospectively tested lentil varieties with high-quality parameters can be utilized by breeders with the idea of exploring high-quality lentil varieties.

## Materials and methods

In the current study, lentil germplasm (100 genotypes), comprising local and exotic genotypes, was collected from different agro-morphological zones of Pakistan. These genotypes were studied for the assessment of different biochemical and nutritional parameters (Table 1). The two-field experiments were carried out at the Nuclear Institute for Agriculture and Biology (NIAB), Faisalabad, Pakistan (longitude 73.08969000 and latitude 31.41554000 with an average rainfall of 526 mm) field area during cropping seasons 2020 and 2021 in a duplicate manner under Randomized Complete Block Design (RCBD), following the same agronomic practices throughout the growth period. The size of the plot was 2 M × 0.30 M, and the plant-to-plant and row-to-row distances were 10 cm and 30 cm.

**Table 1:** List of lentil genotypes (100) used for biochemical analysis in the current study.

Sr. No.	Genotype names	Biological nature	Agronomic characteristics	Origin/source
1.	NLM 15011	M	High biomass, low-yielding mutant	NIAB, Faisalabad
2.	NLM 15012	M	Low biomass, low-yielding mutant	NIAB, Faisalabad
3.	NLM 15014	M	Early mutant	NIAB, Faisalabad
4.	NLM 15015	M	Tall heighted, high pod-bearing mutant	NIAB, Faisalabad
5.	NLM 15016	M	Low-yielding mutant	NIAB, Faisalabad
6.	NLM 15018	M	Tall heighted mutant	NIAB, Faisalabad
7.	NLM 15019	M	High biomass, low pod-bearing mutant	NIAB, Faisalabad
8.	NLM 15020	M	Medium heighted, low-yielding mutant	NIAB, Faisalabad
9.	NLM 15021	M	High biomass and high pod-bearing mutant	NIAB, Faisalabad
10.	NLM 15025	M	High-yielding mutant	NIAB, Faisalabad
11.	NLM 15026	M	High-yielding mutant	NIAB, Faisalabad
12.	NLM 15029	M	High pod-bearing mutant	NIAB, Faisalabad
13.	NLM 15035	M	High pod-bearing and low-yielding mutant	NIAB, Faisalabad
14.	LPP 11001	R	Multiple resistance against fungal diseases	NIAB, Faisalabad
15.	LPP 11025	R	Tall heighted and high pod bearer	NIAB, Faisalabad
16.	LPP 11111	R	Tall heighted and low biomass	NIAB, Faisalabad
17.	LPP 11137	R	Low yield	NIAB, Faisalabad
18.	LPP 11145	R	High biomass and low yield	NIAB, Faisalabad
19.	LPP 11168	R	Tall heighted and high pod bearer	NIAB, Faisalabad
20.	LPP 11195	R	High pod bearer and low yield	NIAB, Faisalabad
21.	LPP 11224	R	Tall heighted and low biomass	NIAB, Faisalabad
22.	LPP 12051	R	High yielding, resistant	NIAB, Faisalabad
23.	LPP 12052	R	High yielding, resistant	NIAB, Faisalabad
24.	LPP 12062	R	High yielding, resistant	NIAB, Faisalabad
25.	LPP 12068	R	High yielding, resistant	NIAB, Faisalabad
26.	LPP 12103	BCR	High yielding	NIAB, Faisalabad
27.	LPP 12105	BCR	High yielding and resistant	NIAB, Faisalabad
28.	LPP 12110	BCR	High yielding and resistant	NIAB, Faisalabad
29.	LPP 12137	R	High yielding and resistant	NIAB, Faisalabad
30.	LPP 12182	R	Tall heighted and high pod bearer	NIAB, Faisalabad
31.	NLH 12088	R	High pod-bearing recombinant	NIAB, Faisalabad
32.	NLH 12096	BCR	Tall heighted and high pods bearer	NIAB, Faisalabad
33.	NLH 11211	R	Medium heighted, and high pods bearer	NIAB, Faisalabad
34.	NLH 11220	R	High pods bearer	NIAB, Faisalabad
35.	NLH 11229	R	Tall heighted and high pod bearing recombinant	NIAB, Faisalabad
36.	NLH 12097	BCR	Tall heighted and high pod bearing recombinant	NIAB, Faisalabad
37.	NLH 12159	R	High yielding, recombinant	NIAB, Faisalabad
38.	NLH 12187	R	Tall heighted and high pod bearing recombinant	NIAB, Faisalabad
39.	NLH 12196	R	Early recombinant	NIAB, Faisalabad
40.	NLH 15003	R	High yielding recombinant	NIAB, Faisalabad
41.	NLH 15032	R	Low biomass, tall heighted, high yielding recombinant	NIAB, Faisalabad
42.	NLH 17026	R	Low biomass, high-yielding recombinant	NIAB, Faisalabad
43.	NLH 17034	R	Early like NIAB Masoor2002	NIAB, Faisalabad
44.	NLH 17039	R	Bold-seeded, high-yielding recombinant	NIAB, Faisalabad
45.	NLI 15044	EG-I	Red podded	ICARDA
46.	NLI 17001	EG-I	Resistant	ICARDA
47.	NLI 17002	EG-I	Early maturing	ICARDA
48.	NLI 17003	EG-I	Heavy biomass	ICARDA
49.	NLI 17057	EG-I	Wild cross	ICARDA
50.	NLI 17058	EG-I	Wild cross	ICARDA
51.	NLI 17059	EG-I	Wild cross	ICARDA
52.	LHM 17006	H/M	High yielding and resistant	NIAB, Faisalabad
53.	LHM 17008	H/M	High yielding and resistant	NIAB, Faisalabad
54.	LHM 17009	H/M	High yielding and resistant	NIAB, Faisalabad
55.	LHM 17010	H/M	High yielding and resistant	NIAB, Faisalabad
56.	LHM 17011	H/M	High yielding and resistant	NIAB, Faisalabad
57.	LHM 17013	H/M	High yielding and resistant	NIAB, Faisalabad
58.	LHM 17014	H/M	High yielding and resistant	NIAB, Faisalabad
59.	LHM 17015	H/M	High yielding and resistant	NIAB, Faisalabad
60.	LHM 17019	H/M	High yielding and resistant	NIAB, Faisalabad
61.	LHM 17020	H/M	High yielding and resistant	NIAB, Faisalabad
62.	LHM 17021	H/M	High yielding and resistant	NIAB, Faisalabad
63.	LHM 17022	H/M	High yielding and resistant	NIAB, Faisalabad
64.	LHM 17023	H/M	High yielding and resistant	NIAB, Faisalabad
65.	LHM 17024	H/M	High yielding and resistant	NIAB, Faisalabad
66.	Masoor 85	AV	Light green, medium heighted	AARI, Faisalabad
67.	Mansehra-89	AV	High yielding, medium heighted, and blight resistant	NARC, Islamabad
68.	Masoor 93	AV	Medium green, spreading	AARI, Faisalabad
69.	Shiraz-96	AV	Green, erect	AZRC, Quetta
70.	NIAB Masoor 2002	AV	Early, rust resistant	NIAB, Faisalabad
71.	NIA Masoor 05	AV	Low biomass	NIA, Tandojam
72.	NIAB Masoor 2006	AV	Medium, high yield, and small seeded	NIAB, Faisalabad
73.	Markaz 2009	AV	Green, erect	NARC, Islamabad
74.	Punjab Masoor 2009	AV	High yielding, medium heighted, and lodging resistant	AARI, Faisalabad
75.	Chakwal Masoor	AV	Light-colored seed	BARI, Faisalabad
76.	Punjab Masoor 2019	AV	High yield, rust resistant	AARI, Faisalabad
77.	Punjab Masoor 2020	AV	High yielding, disease-resistant	NIAB & AARI, Faisalabad
78.	NL 96621	OG	Very dark, more no. of primary branches, semi-erect	NIAB, Faisalabad
79.	NL 96635	OG	Green, spreading	NIAB, Faisalabad
80.	NL 96680	OG	Late, very dark, spreading, and branching	NIAB, Faisalabad
81.	NL 96700	OG	Green, spreading	NIAB, Faisalabad
82.	Turk Masoor	EG-I	Wilt disease susceptible	Turkey
83.	Lentil Black	EG-I	Black-colored seed	ICARDA
84.	TCL 85–1	M	Tissue culture line	NIAB, Faisalabad
85.	ALTINOPARK	EG-I	Herbicide-tolerant	ICARDA
86.	SEYRAN-96	EG-I	Herbicide-tolerant	ICARDA
87.	GAEIL	EG-I	Herbicide-tolerant	ICARDA
88.	ILL 18108	EG-I	Low biomass	ICARDA
89.	Precoz	EG-I	Mutant, large-seeded, rust resistant	ICARDA
90.	ILL 6002	EG-I	Heat tolerant	ICARDA
91.	ILL 2245	EG-I	Low yielding	ICARDA
92.	ILL 2580	EG-I	High yielding	ICARDA
93.	ILL 4400	EG-I	Wilt resistant	ICARDA
94.	ILL 8006	EG-I	Early maturing	ICARDA
95.	ILL 7978	EG-I	Stemphylium blight resistant	ICARDA
96.	X 2013–174-1	EG-I	Low no. of secondary branches	ICARDA
97.	X 2011S-160-22	EG-I	Heavy biomass	ICARDA
98.	X 2011S 33–34-32	EG-I	High pods bearing	ICARDA
99.	X 2011S-19-12	EG-I	Medium heighted	ICARDA
100.	X 2011S-19-39	EG-I	High pods bearing	ICARDA

M: mutant, EG-I: exotic genotype-introduction, R: recombinant, AV: approved variety, BCR: back cross recombinant, OG: old genotype, H/M: hybrid mutant.

Lentil germplasm (100 genotypes) was harvested during 2020 and 2021 for biochemical and nutritional analysis (Table 1). The seed samples were packed in plastic bottles to ensure integrity. The seed analysis was performed in Marker-Assisted Breeding (MAB) Lab-1, Plant Breeding and Genetics Division (PBGD), Nuclear Institute of Agriculture and Biology (NIAB), Faisalabad, Pakistan.

## Sample extraction and preparation

Lentil seeds were ground to a fine powder in a pestle mortar, and then 1 mL of 50 mM potassium phosphate buffer (KH_2_PO_4_) was added to the 0.1 g flour sample. Samples were vortexed and centrifuged at 14,462× *g* for 10 min at 4°C for homogenization. The supernatant was taken for evaluation of non-enzymatic and enzymatic antioxidant activities and other biochemical and nutritional parameters.

## Non-enzymatic antioxidant

### Ascorbic acid

For the determination of ascorbic acid content in lentil seed samples, the dichloroindophenol (DCIP) method was used as described by Hameed et al. (26). In this assay, the reaction mixture contained 110 μL of DCIP (0.2 mg DCIP per ml of distilled water), 110 μL of 0.1% meta phosphoric acid, 100 μL sample extract, and 900 μL distilled water.

We measured reduced ascorbic acid only with this method. Shortly, ascorbic acid converts the DCIP molecule to its reduced form, DCIPH2, which can be monitored by measuring the decrease in absorbance at 520 nm spectrophotometrically.

### Total phenolic content

A colorimetric method was used to determine the total phenolic content, which was described by Ainsworth and Gillespie (27) with slight modifications. In this analysis, 0.45 g of ground seed samples were homogenized in 500 μL of ice-cold 95% methanol and then incubated at room temperature for 48 h in the dark. Then the samples were centrifuged at 14,462× *g* for 5 min at room temperature. The supernatant was used for measuring TPC content in samples. A 150 μL supernatant was mixed with 150 μL 10% (v/v) F-C reagent, vortexed, and then 1,200 μL of 700 mM Na_2_CO_3_ was added. The sample mixture was incubated at room temperature for 1 h. Then the blank corrected absorbance was noted at 765 nm.

### Tannins

Tannins were calculated from seed samples by following the method of Ainsworth and Gillespie (27). Polyvinylpolypyrrolidone (PVPP) (0.1 g) was added to the ground sample and centrifuged at 14,462× *g* for 10 min. The absorbance of the supernatant was measured at 765 nm by using a spectrophotometer (Hitachi) model number U-2800.

## Enzymatic antioxidants

### Catalase (CAT) activity

Catalase activity of the samples was measured using the method of Beers and Sizer (28). In this method, ground seeds were homogenized in 50 mM potassium phosphate buffer (pH 7.0). The assay solution consisted of 2 mL of 50 mM phosphate buffer (pH 7.0), 100 μL of 59 mM H_2_O_2_, and 0.1 mL of enzyme extract. The decrease in absorbance pattern was recorded after every 20 s at 240 nm. 1 U of enzyme activity was expressed as 0.01 absorbance change min^−1^, and the activity of each enzyme was checked on the basis of seed weight.

### Peroxidase activity

For the evaluation of peroxidase (POD) activity, ground seed samples were homogenized in 50 mM potassium phosphate buffer (pH 7.0). Peroxidase activity was determined by using the modified method of Chance and Maehly (29). For the preparation of the assay solution, distilled water (535 μL) was added to guaiacol (100 μL), H_2_O_2_ (100 μL), potassium phosphate buffer (pH 7.0), and enzyme extract (15 μL). The reaction was initiated after adding the enzyme extract to the sample solution. The increase in absorbance of the sample solution was noted every 20 s at 470 nm. 1 U of POD activity was termed as 0.01 absorbance change min^−1^, and enzyme activity was checked on the basis of seed weight.

### Ascorbate peroxidase activity

For the determination of ascorbate peroxidase (APX) activity, ground seed samples were homogenized in 50 mM potassium phosphate buffer (pH 7.0). APX activity was recorded using the method described by Dixit et al. (30). Assay buffer was made by adding ascorbic acid (3.4 mL), 0.5 M EDTA (10 mL), and 0.2 M potassium phosphate buffer (pH 7.0). For the preparation of the reaction mixture, assay buffer (1 mL) was added to 4 M H_2_O_2_ (1 mL) and enzyme extract (50 μL). The rate of ascorbic acid oxidation was checked by measuring the decrease in absorbance at 290 nm every 30 s.

### Superoxide dismutase activity

The activity of superoxide dismutase was measured by homogenizing ground seeds in 50 mM potassium phosphate buffer (pH 7.0) and 0.1 mM EDTA, as demonstrated by Dixit et al. (30). The assay buffer was prepared by mixing distilled water (400 μL), 200 mM potassium phosphate buffer pH 7.0 (250 μL), l-methane (100 μL), Triton X-100 (100 μL), NBT (50 μL), enzyme extract (50 μL), and riboflavin (50 μL). SOD activity was checked by measuring the photochemical reduction of nitroblue tetrazolium (NBT). 1 U of enzyme activity of SOD is expressed as the amount of enzyme responsible for the inhibition of photochemical reduction of NBT by 50%.

## Hydrolytic enzymes

### Alpha-amylase activity

The alpha-amylase activity of seed samples was determined using the method described by Varavinit (31). In this method, there are two reagents: 3,5 dinitrosalicylic acid (DNS) and 1% starch solution. The DNS solution was prepared by adding 96 mM DNS (1 g DNS in 50 mL of distilled water) + sodium potassium tartrate (30 g) + 2 N NaOH (20 mL) and making the final volume 100 mL by using distilled water. The reaction mixture contained 0.2 mL of sample, 1.8 mL of distilled water, and 1 mL of 1% starch solution. The reaction mixture was incubated for 3 min. Then 1 mL of DNS reagent was added to each sample mixture, placed in water for 15 min at 100°C, and allowed to cool to room temperature. Finally, 9 mL of distilled water was added to each sample mixture, and the absorbance was measured at 540 nm using a spectrophotometer.

### Protease activity

To determine protease activity, seed samples were homogenized in 50 mM potassium phosphate buffer (pH 7.8). Protease activity was determined with the casein digestion assay elaborated by Drapeau (32). According to this method, 1 U of protease activity is the amount of enzyme that generates acid-soluble portions equivalent to 0.001 A280/min at 37°C with a pH of 7.8.

## Other biochemical aspects

### Malondialdehyde content (MDA)

Lipid peroxidation activity of an enzyme can be determined by the malondialdehyde content present in it. MDA activity was measured using the method of Heath and Packer, with slight modifications elaborated by Dhindsa (33). The seed sample (0.25 g) was added to 0.1% TCA. The sample mixture was centrifuged at 14,462× *g* for 5 min. Then 20% TCA and 0.05% TBA (solution) were added to the supernatant. The reaction mixture was heated at 95°C for 30 min and cooled down immediately in an ice bath. Now the samples were centrifuged for 10 min at 14,462× *g*. The absorbance of the supernatant was noted at 532 nm, and non-specific absorption values were checked at 632 nm and subtracted. The MDA content in seed samples was measured in terms of an MDA coefficient of 155 m/M/cm.

### Total oxidant status

Total oxidant status of seed samples was measured using the method of Erel (34). The amount of oxidants present in the samples, which convert ferrous ions to ferric ions in an acidic medium, was measured by using xylenol orange. In this assay, reagent 1 + reagent 2 and sample extract were used. The reagent R1 was the stock xylene orange solution containing 75 μL xylenol orange dye (0.38 g xylenol orange in 500 μL of 25 mM H_2_SO_4_), 0.409 g of NaCl, 500 μL of glycerol, and the final volume was made up to 50 mL with 25 mM H_2_SO_4_. The reagent 2 (R2) contained 0.0317 g of o-dianisidine and 0.0196 g of ferrous ammonium sulfate in 10 mL of 25 mM H_2_SO_4_. After adding 900 μL of reagent 1, 140 μL of sample, and 44 μL of reagent 2, the reaction mixture was incubated for 5 min. Then the absorbance of the reaction mixture was measured at 560 nm by using a spectrophotometer. After that, a standard curve for H_2_O_2_ was prepared. The results for TOS were expressed in μM H_2_O_2_ eq/L.

### Protein content

For protein content determination, seed samples were added to 50 mM potassium phosphate buffer at pH 7. Quantitative protein was determined by using the method of Bradford (35). To perform this assay, a sample mixture was prepared by adding the supernatant (5 μL) with 0.1 N NaCl and 1 mL Bradford dye and incubating them for 15 min to form a dye–protein complex. Then the absorbance was checked at 595 nm with a spectrophotometer.

### Total soluble sugars (reducing and non-reducing sugars)

Total soluble sugars in seed samples were determined by using the method of Dubois et al. (36) with phenol–sulfuric acid reagent. The amount of reducing sugars present in the samples was determined by the method of dinitrosalicylic acid by Miller (37), and non-reducing sugars were calculated by the difference between total soluble sugars and reducing sugars.

### Total antioxidant capacity

Total antioxidant capacity (TAC) of lentil seed samples was measured using the method of Erel (34) with some necessary modifications. Reagent 1 (sodium acetate trihydrate) 0.16 g/150 mL and reagent 2 were made by mixing 30 mM Na-acetate, glacial acetic acid, H_2_O_2_, and ABTS solution 2 mM/L. In this assay, the reduced molecule of ABTS is oxidized to ABTS^•+^ by using hydrogen peroxide in acidic medium. Reduced ABTS molecules are stable for a long time, but they change their color when diluted with more acetate solutions with high pH values. The strength of the reduced ABTS molecules was checked at 660 nm spectrophotometrically.

### Total flavonoids

Total flavonoid content in lentil seed samples was determined by using the aluminum chloride colorimetric method of Lin and Tang (38). In this procedure, 0.1 mL of 10% aluminum chloride, 0.1 M potassium acetate, and 2.8 mL of deionized water were added to the 2 mL sample. Then the reaction mixture was incubated for 40 min at ambient temperature. After incubation, the absorbance of the reaction mixture was measured at 415 nm spectrophotometrically.

### Pigment analysis

Seed samples (0.075 g) were ground in a pestle mortar, and 1.5 mL of 80% acetone was added. These sample mixtures were then placed in the dark for overnight. Samples were centrifuged at 14,462× *g* for 10 min at 4°C. Finally, absorbance for carotenoids, chlorophyll a, chlorophyll b, lycopene, and total chlorophyll were recorded at 663 nm, 645 nm, 505 nm, 470 nm, and 453 nm, respectively, on the HITACHI Spectrophotometer (U-2800). Pigments (lycopene, carotenoids, chl. a, chl. B, and total chlorophyll) were measured by the methods of Arnon (39) and Lichtenthaler and Wellburn (40).

### Statistical analysis

The data obtained from the current study were subjected to different statistical analyses using XL-STAT software version 2020.5. In order to organize the resulting data, descriptive statistics were performed. Correlation study, analysis of variance (ANOVA) (41) and Tukey HSD test at *p* < 0.05 were done to check the significance of the resulting data. Mean ± standard errors were used to construct graphs. For the selection of reliable genotypes, principal component analysis (PCA) and cluster analysis were also performed by algometric hierarchical clustering of the tested genotypes with the help of XL-STAT software version 2020.5 (42).

## Results

### Classification of germplasm

A total of 100 lentil genotypes used in this study were divided into three categories (low, medium, and high). The classification of genotypes was performed on the basis of different comparative values obtained for each studied biochemical parameter (Supplementary Table S1). Descriptive statistics of the groups of genotypes in the current study were also performed (Supplementary Table S2). The groupwise comparison using analysis of variance (ANOVA) and the LSD test was mentioned in Tables 2A,B.

**Table 2:** Analysis of variance (ANOVA) and LSD for group-wise comparison of the lentil genotypes used in the current study.

A											
Category	Tanins (mean)	AsA (mean)	CAT (mean)	APX (mean)	TPC (mean)	POD (mean)	MDA (mean)	TOS (mean)	TS (mean)	RS (mean)	NRS (mean)
BCR	8797.500 a	638.450 a	1925.000 a	749.500 ab	31496.000 ab	827.390 a	222.923 ab	3395.200 bc	43.444 a	5.551 ab	38.003 a
AV	8003.333 a	605.917 a	676.292 b	786.458 ab	32428.542 a	505.246 ab	224.136 a	2116.042 c	40.504 a	5.259 b	35.775 a
OG	9136.875 a	617.500 a	598.500 b	974.375 a	32221.250 ab	130.313 b	210.996 abc	345.250 c	40.178 a	5.229 b	37.249 a
EG-I	10295.020 a	636.850 a	800.280 b	694.480 ab	23938.800 bc	371.255 ab	200.223 abc	9974.200 ab	40.939 a	12.192 a	29.094 a
M	7805.000 a	634.918 a	417.321 b	514.750 b	18540.071 cd	385.200 ab	187.424 bc	12971.786 a	44.803 a	9.069 ab	36.217 a
R	9113.077 a	632.827 a	857.835 b	603.962 ab	19603.538 cd	252.975 b	195.980 bc	7264.654 b	44.247 a	9.755 ab	33.910 a
H/M	8930.179 a	632.482 a	415.214 b	720.179 ab	15963.750 d	93.557 b	179.745 c	10610.643 ab	40.328 a	10.606 ab	29.230 a
Pr > F (Model)	0.550	0.358	0.085	0.249	0.002	0.140	0.037	0.001	0.937	0.114	0.579
Significant	No	No	No	No	Yes	No	Yes	Yes	No	No	No

B											
Category	Protease (mean)	SOD (mean)	TSP (mean)	Amylase (mean)	TAC (mean)	LYCO (mean)	CHLA (mean)	CHLB (mean)	TOTAL CAR (mean)	TOTAL CHLORO (mean)	TF (mean)
BCR	10892.700 abc	219.834 a	184.787 c	114.526 a	4.882 ab	5.132 ab	116.100 ab	126.366 ab	9.235 ab	241.918 ab	337.366 a
AV	12050.833 a	141.595 b	289.981 abc	111.609 a	5.735 ab	7.572 a	151.055 a	197.235 a	13.738 a	349.793 a	289.508 ab
OG	12031.875 ab	116.033 b	239.207 bc	116.153 a	6.049 a	7.503 a	151.154 a	184.361 a	13.506 a	329.037 a	302.691 a
EG-I	9904.320 bc	193.614 a	338.797 ab	100.651 a	5.147 ab	3.652 b	80.858 bc	88.425 b	6.869 b	168.151 b	209.931 bc
M	8696.964 c	188.300 a	378.557 a	98.682 a	5.842 ab	3.495 b	81.273 bc	96.437 b	6.506 b	172.575 b	153.051 c
R	9092.154 c	214.361 a	346.785 ab	98.176 a	2.707 b	3.452 b	75.543 bc	84.266 b	6.633 b	160.612 b	177.266 c
H/M	8794.107 c	186.987 a	369.400 ab	112.650 a	4.377 ab	2.695 b	63.561 c	73.245 b	4.923 b	136.584 b	159.389 c
Pr > F (Model)	0.000	0.001	0.033	0.627	0.077	<0.0001	<0.0001	0.000	<0.0001	<0.0001	<0.0001
Significant	Yes	Yes	Yes	No	No	Yes	Yes	Yes	Yes	Yes	Yes

## Non-enzymatic antioxidants

### Ascorbic acid

The high class of ascorbate ranged from 681 to 706 μg/g s. wt. and included only five genotypes, and the highest value of ascorbic acid was found in LPP 12182 (706 μg/g s. wt.). Twenty-eight genotypes were observed in the medium class, ranging from 641 to 680 μg/g s. wt., and 67 genotypes were grouped in the low class, having a low ascorbic acid content ranging from ≤640 μg/g s. wt. Among these genotypes, the lowest ascorbic acid content was found in Shiraz-96 (333 μg/g s. wt.) (Supplementary Figure S1A).

### Total phenolic content

Genotypes with high class ranged from 30,001 to 54,600 μM/g s. wt. and included 31 genotypes. The highest TPC content was found in NLI 17003 (54,600 μM/g s. wt.). Fourteen genotypes were classified in the intermediate class, ranging from 15,001 to 30,000 μM/g s. wt. Low levels were recorded in 55 genotypes with values ≤15,000, and the lowest value was noticed in LHM 17010 (4,755 μM/g s. wt.) (Supplementary Figure S1B).

### Tannins

High class of tannins included 42 genotypes, ranging from 10,000 to 25,000 μM/g s. wt., and NLI 17057 (24,563 μM/g s. wt.) acquired the higher tannin content. Furthermore, 44 genotypes were assembled in the medium class, ranging from 5,001 to 9,999 μM/g s. wt. Genotypes having tannin values ≤5,000 were placed in the low class and consisted of 14 genotypes, and the lowest value of tannins was noticed in LPP 11137 (257 μM/g s. wt.) (Supplementary Figure S2A).

## Enzymatic antioxidants

### Catalase (CAT) activity

For high-class catalase, only 10 genotypes were recorded, ranging from 2001 to 5,600, and the highest value was found in LPP 12110 (5,595 Units/g s. wt.). Eleven genotypes were classified in the medium class, ranging from 1,001 to 2000. Seventy-nine genotypes expressed low catalase activity, and NLM 15019 (30 Units/g s. wt.) showed the lowest catalase activity (Supplementary Figure S2B).

### Ascorbate peroxidase activity

Fourteen genotypes were grouped in high class, ranging from 1,001 to 2,500 Units/g s. wt. and the highest activity was found in ALTINOPARK (2,465 Units/g s. wt.). Intermediate class included 26 genotypes, ranging from 701 to 1,000 Units/g s. wt. Low levels ≤700 Units/g s. wt. were observed in 60 genotypes, and the lowest value was noticed in NLM 15011 and X 2013-174-1 (41.500 Units/g s. wt.) (Supplementary Figure S3A).

### Peroxidase activity

High levels were recorded in only six genotypes, ranging from 1,501 to 3,200 Units/g s. wt., and the highest activity was shown by NIAB Masoor 2002 (3,170 Units/g s. wt.). Twelve genotypes were assembled in the medium class, ranging from 501 to 1,500 Units/g s. wt. Moreover, low levels were observed in 82 genotypes, range ≤ 500 Units/g s. wt. Among the tested genotypes, the lowest activity was noticed in LPP 12062 (Supplementary Figure S3B).

### Superoxide dismutase activity

High level comprised 43 genotypes, ranging from 201 to 296 Units/g s. wt., and the highest value was noticed in LPP 11195 (294.096 Units/g s. wt.). On the other hand, 33 genotypes were placed in the medium class, ranging from 141 to 200 Units/g s. wt. Twenty-four genotypes were assembled in low class for SOD content range ≤ 140 Units/g s. wt. and the least value was observed in LHM 17019 (66.97 Units/g s. wt.) (Supplementary Figure S4A).

## Hydrolytic enzyme activity

### Alpha-amylase activity

Only four genotypes showed high alpha-amylase content ranging from 151 to 273 mg/g s. wt., and the highest value was found in LPP 12137 (271.87 mg/g s. wt.). Furthermore, the medium class included 54 genotypes ranging from 101 to 150 mg/g s. wt. Low levels were noticed in 42 genotypes ranging ≤100 mg/g. s. wt., and NLM 15035 (60.54 mg/g. s. wt.) exhibited the lowest activity (Supplementary Figure S4B).

### Protease activity

Among all tested genotypes, the high class included 40 genotypes ranging from 10,001 to 17,000 Units/g. s. wt., and Masoor 93 exhibited the highest value (16,050 Units/g. s. wt.). Thirty-three genotypes exhibited intermediate protease content ranging from 8,001 to 10,000 Units/g. s. wt. Low levels were recorded in 27 genotypes ranging ≤8,000 Units/g. s. wt., and LPP 11111 (6,125 Units/g. s. wt.) showed the lowest protease content (Supplementary Figure S5A).

## Other biochemical parameters

### Total oxidant status

High class included only 17 genotypes ranging from 15,001 to 48,000 μM/g. s. wt., and the highest value was observed in NLM 15029 (47,487 μM/g. s. wt.). Among all genotypes, 40 genotypes were assorted in the medium class, ranging from 5,001 to 15,000 μM/g. s. wt. Forty-three genotypes exhibited low levels ranging ≤5,000 μM/g. s. wt., and the lowest value of TOS was recorded in NLI 17057 (48 μM/g. s. wt.) (Supplementary Figure S5B).

### Malondialdehyde content

Twenty-four genotypes were grouped into high class, ranging from 231 to 301 Units/g s. wt., and the highest value was found in the local Pakistani variety Punjab Masoor, 2009 (300.5 μM/g s. wt.). Medium class included a larger number of genotypes, and 60 genotypes were assembled in this group, ranging from 161 to 230 μM/g s. wt. On the other hand, low MDA content was noticed in 16 genotypes ranging ≤160 μM/g s. wt., and the lowest value was shown by NLH 17034 (97.82 μM/g s. wt.) (Supplementary Figure S6).

### Protein content

High protein levels were found in 58 genotypes, ranging from 351 to 548 mg/g s. wt. Highest value was found in X 2011S-19-39 (546.33 mg/g s. wt.). Eighteen genotypes were placed at medium levels ranging from 201 to 350 mg/g s. wt. Low level was found in 24 genotypes under the range ≤ 5 mg/g s. wt., and ILL 18108 exhibited the lowest protein content (38.17 mg/g s. wt.) (Supplementary Figure S7A).

### Total soluble sugars content

Twenty genotypes were included in the high class, ranging from 51 to 85 mg/g. s. wt., and NLM 15015 (83.9 mg/g. s. wt.) possessed the highest soluble sugar content. On the other hand, 64 genotypes were grouped in the medium class, ranging from 31 to 50 mg/g. s. wt. In the low class, 16 genotypes were found, and the lowest value was noticed in GAEIL (11.49 mg/g. s. wt.) (Supplementary Figure S7B).

### Reducing sugar content

Only three genotypes were classified in the high class of reducing sugars, ranging from 30.1 to 46 mg/g. s. wt., and the highest content was found in ILL 8006 (45.68 mg/g. s. wt.). While 21 genotypes were categorized in the medium class, ranging from 10.1 to 30 mg/g. s. wt. Seventy-six genotypes were placed in the low class under the range ≤ 10 mg/g. s. wt., and the lowest RS value was noticed in Markaz 2009 (3.0 mg/g. s. wt.) (Supplementary Figure S8A).

### Non-reducing sugars content

Only seven genotypes exhibited high NRS values, ranging from 50.1 to 75 mg/g. s. wt., and NLM 15015 (74.7 mg/g. s. wt.) exhibited the highest NRS values. Seventy-two genotypes were categorized in the medium class, ranging from 20.1 to 50 mg/g s. wt. Among all tested genotypes, low values were observed in 21 genotypes ranging ≤20 mg/g. s. wt., and X 2011S-160-22 (3.69 mg/g s. wt.) exhibited the least NRS content (Supplementary Figure S8B).

### Total antioxidant capacity

In the high category, eight genotypes were observed, ranging from 10.1 to 16 μM/g. s. wt., and NLM 15016 showed the highest TAC value (15.76 μM/g. s. wt.). Thirty-seven genotypes were classified in the medium class, ranging from 5.01 to 10 μM/g. s. wt. Fifty-five genotypes were placed in the low class, ranging ≤5 μM/g. s. wt., and the lowest value was observed in LPP 11224 (0.331 μM/g s. wt.) (Supplementary Figure S9A).

### Total flavonoid content

Thirty-four genotypes were assembled in high class, ranging from 300.01 to 367 μg/mL s. wt., and the highest value was noticed in NLI 17059 (366.42 μg/mL s. wt.). Medium class included 14 genotypes ranging from 150.01 to 300 μg/mL s. wt. Fifty-two genotypes were grouped in low class, ranging ≤150 μg/mL s. wt., and NLH 12187 showed the least TF value (57.36 μg/mL s. wt.) (Supplementary Figure S9B).

### Pigments content

Thirty-four genotypes were assorted in high class, ranging from 10.01 to 18 μg/g s. wt., and the highest value was exhibited by Markaz 2009 (17.89 μg/g s. wt.). Medium class included 22 genotypes ranging from 5.01 to 10 μg/g s. wt. Low carotenoid content was observed in 44 genotypes ranging ≤5 μg/g s. wt., and the lowest value was found in ILL 6002 (1.31 μg/g s. wt.) (Supplementary Figure S10A).

Only three genotypes were grouped in high class, ranging from 200.01 to 240 μg/g s. wt., and the highest (chlorophyll a) content was observed in NLH 12097 (236.12 μg/g s. wt.). In the medium class, 40 genotypes were included, ranging from 100.01 to 200 μg/g s. wt. Among all tested genotypes, 57 genotypes were found in the low class, ranging ≤100 μg/g s. wt., and LHM 17010 (25.14 μg/g s. wt.) possessed the least value (Supplementary Figure S10B).

Sixteen genotypes are assembled in high class, ranging from 200.01 to 320 μg/g s. wt., and the highest chlorophyll b content was found in NLH 12097 (317 μg/g s. wt.). Medium class included 31 genotypes ranging from 100.01 to 200 μg/g s. wt. Low levels of chlorophyll b included 53 genotypes, and NLH 11211 (8.39 μg/g, s. wt.) showed the lowest chlorophyll b content (Supplementary Figure S11A).

The highest value of total chlorophyll was found in NLH 12097 (552.58 μg/g s. wt.). Medium class included 27 genotypes ranging from 150.01 to 250 μg/g s. wt. Furthermore, 41 genotypes were classified in the low class, ranging ≤150 μg/g s. wt., and the lowest value was noticed in NLH 11211 (35.19 μg/g s. wt.) (Supplementary Figure S11B).

Only three genotypes were placed in high class, ranging from 10.00 to 11.00 μg/g s. wt., and NLH 12097 (10.881 μg/g s. wt.) showed the highest lycopene content. In the medium class, 30 genotypes were assembled, ranging from 5.0 to 9.99 μg/g s. wt. On the other hand, low lycopene values were observed in 67 genotypes ranging ≤5 μg/g s. wt., and NLH 11211 (0.686 μg/g s. wt.) exhibited the least value (Supplementary Figure S12).

### Principal component analysis

**Figure 1:**
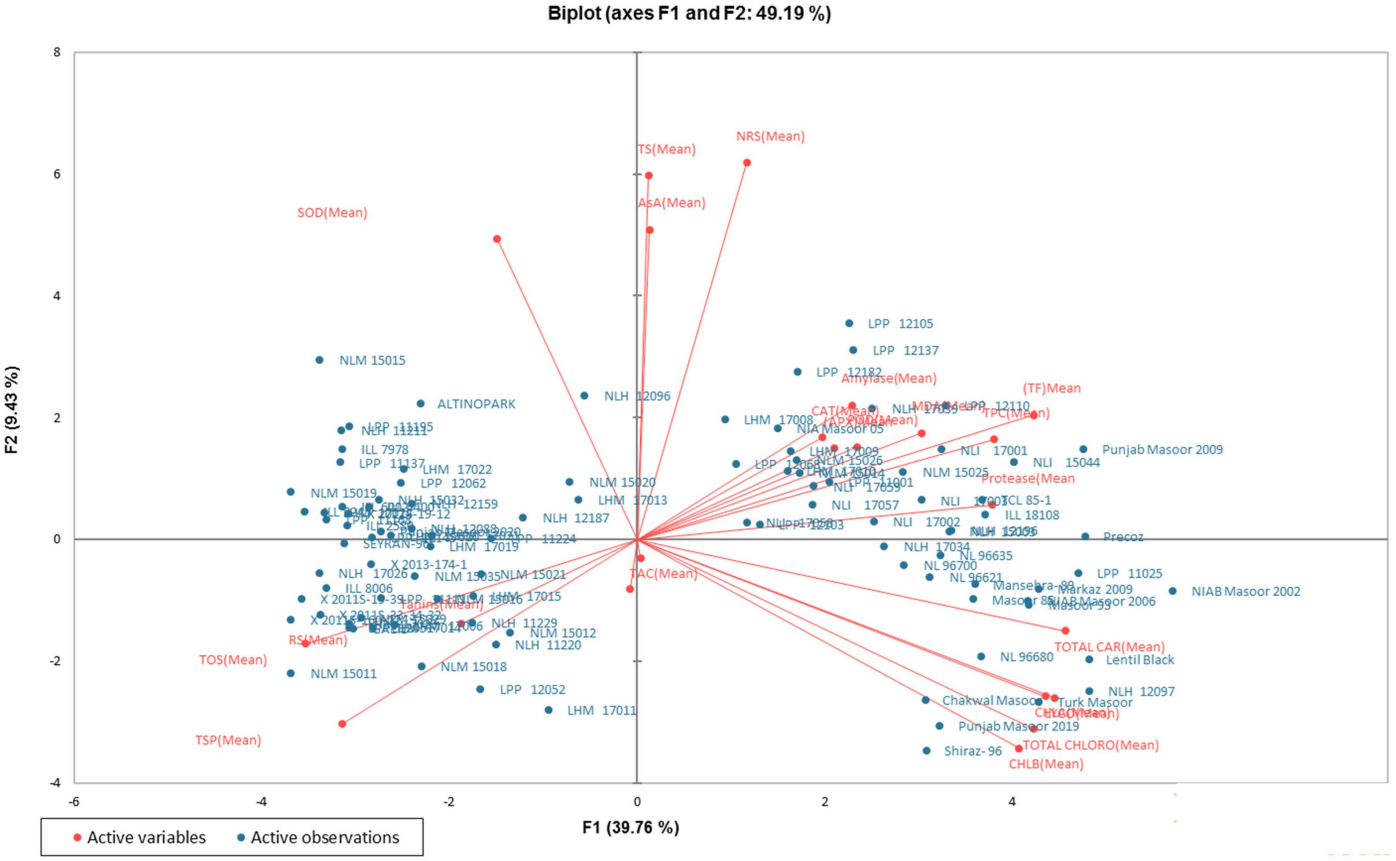
Genotype-by-trait biplot of lentil genotypes for the first two PCs (principal components).

In order to analyze the variation among the tested genotypes, the data were transformed into principal factors. The analyzed data were subjected to principal component analysis (PCA) to investigate the interrelationship between variables. Out of 22 (Pc) principal components, five PCs: PC-1, PC-II, PC-III, PC-IV, and PC-V, showed Eigenvalues >1 and exhibited 69.23% cumulative variability among 100 lentil genotypes (Table 3). The contribution of PC-1 toward variability was highest (39.75%), followed by PC-II (9.43%), PC-III (8.15%), PC-IV (6.33%), PC-V (5.55%), PC-VI (4.48%), and PC-VII (3.69%). The biplot demonstrates the overall association of lentil genotypes for 22 traits (Figure 1). The first two principal components (PCs) that contributed (49.19%) toward the total variance were drawn as PC-1 (39.76%) on the *x*-axis and PC-II (9.43%) on the *y*-axis for the determination of association among different clusters. The genotype-by-trait (G-T) biplot demonstrated 49.19% of total variation. To show the interrelationship between different characters in a genotype by trait biplot, a vector was plotted from the origin toward every trait.

**Table 3:** Principal component analysis for biochemical traits in lentil genotypes.

	F1	F2	F3	F4	F5
Eigenvalue	8.74	2.07	1.79	1.39	1.22
Variability (%)	39.75	9.43	8.15	6.33	5.55
Cumulative %	39.75	49.18	57.33	63.67	69.23
Factor loadings					
Tannin	−0.005	−0.057	−0.275	0.681	0.049
AsA	0.009	0.361	−0.306	0.037	0.229
CAT	0.140	0.119	−0.251	0.146	−0.351
APX	0.149	0.106	−0.143	−0.065	0.311
TPC	0.270	0.116	−0.143	−0.065	0.067
POD	0.167	0.108	−0.255	0.247	0.187
MDA	0.216	0.124	−0.042	−0.126	−0.250
TOS	−0.251	−0.121	0.055	0.49	0.202
TS	0.009	0.424	0.478	0.340	0.009
RS	−0.133	−0.098	−0.090	0.342	0.004
NRS	0.083	0.439	0.504	0.142	0.012
Protease	0.268	0.040	−0.150	−0.120	0.057
SOD	−0.106	0.350	−0.210	0.183	0.012
Protein	−0.223	−0.215	−0.074	0.227	0.048
Amylase	0.163	0.156	−0.044	−0.033	−0.004
TAC	0.003	−0.022	0.071	−0.114	0.751
Lycopene	0.316	−0.185	0.111	0.049	0.001
CHL.A	0.309	−0.183	0.104	0.036	0.066
CHL.B	0.289	−0.244	0.160	0.074	0.057
Carotenoids	0.324	−0.107	0.059	0.015	−0.030
Total CHL.	0.300	−0.221	0.151	0.062	0.055
Total flavonoids	0.300	0.145	−0.095	−0.111	0.024

### Cluster analysis

**Figure 2:**
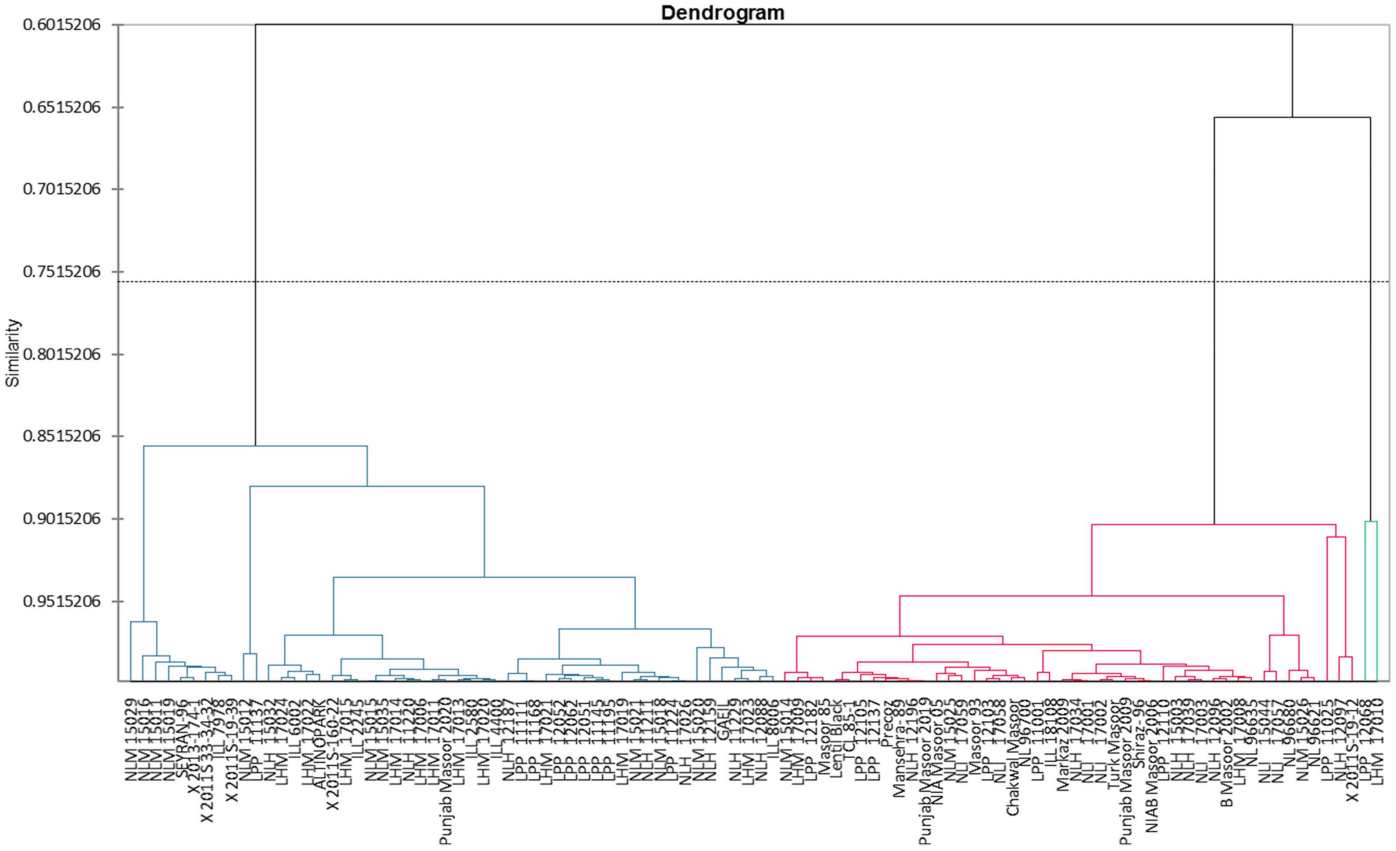
Tree diagram showing the clustering of 22 traits for different lentil genotypes.

Clustering of different studied genotypes based on various traits is shown in Table 4. A total of 100 lentil genotypes are divided into three clusters undergoing cluster analysis (Figure 2). Cluster I exhibited 52 genotypes, followed by cluster II, which encompassed 46 genotypes, and finally, cluster III included only two genotypes, which showed remarkable variability in the different studied traits.

**Table 4:** Distribution of lentil genotypes regarding biochemical characteristics in various clusters.

Cluster	Genotypes
I	NLM 15011, NLM 15012, NLM 15015, NLM 15016, NLM 15018, NLM 15019, NLM 15020, NLM 15021, NLM 15029, NLM 15035, LPP 11111, LPP 11137, LPP 11145, LPP 11168, LPP 11195, LPP 11224, LPP 12051, LPP 12052, LPP 12062, NLH 12088, NLH 11211, NLH 11220, NLH 11229, NLH 12159, NLH 12187, NLH 15032, NLH 17026, LHM 17006, LHM 17011, LHM 17013, LHM 17014, LHM 17015, LHM 17019, LHM 17020, LHM 17021, LHM 17022, LHM 17023, LHM 17024, Punjab Masoor 2020, ALTINOPARK, SEYRAN 96, GAEIL, ILL 6002, ILL 2245, ILL 2580, ILL 4400, ILL 8006, ILL 7978, X 2013–174-1, X 2011S-160-22, X 2011S 33–34-32, X 2011S-19-39
II	NLM 15014, NLM 15025, NLM 15026, LPP 11001, LPP 11025, LPP 12103, LPP 12105, LPP 12110, LPP 12137, LPP 12182, NLH 12096, NLH 12097, NLH 12196, NLH 15003, NLH 17034, NLH 17039, NLI 15044, NLI 17001, NLI 17002, NLI 17003, NLI 17057, NLI 17058, NLI 17059, LHM 17008, LHM 17009, Masoor 85, Mansehra-89, Masoor 93, Shiraz-96, NIAB Masoor 2002, NIA Masoor 05, NIAB Masoor 2006, Markaz 2009, Punjab Masoor 2009, Chakwal Masoor, Punjab Masoor 2019, NL 96621, NL 96635, NL 96680, NL 96700, Turk Masoor, Lentil Black, TCL 85–1, ILL 18108, Precoz, X 2011S-19-12
III	LPP 12068, LHM 17010

Cluster I consisted of genotypes with high mean values of tannin, total oxidant status (TOS), reducing sugars (RS), and protein content. However, cluster II possessed genotypes with high mean values of total phenolic content (TPC), peroxidase (POD), malondialdehyde (MDA) content, total antioxidant capacity (TAC), protease, chlorophyll a, chlorophyll b, total chlorophyll, and carotenoids. Among all clusters, cluster three was comprised of ascorbate (AsA), catalase (CAT), ascorbic peroxidase (APX), alpha-amylase, total soluble sugars (TSS), non-reducing sugars (NRS), superoxide dismutase (SOD), and total flavonoids (Tables 5A,B). Moreover, LPP 12068 and LHM 17010 have been placed in cluster III, showing remarkable differences with cluster II regarding different biochemical traits in Figure 2. In this view, cluster analysis arranged genotypes together, showing similarities among different traits; the grouping of the clusters had not possibly included all genotypes of similar origin.

**Table 5:** Mean values of studied biochemical traits for different lentil genotypes in cluster analysis.

A											
Cluster	Tannins (μM/g)	AsA (μg/g)	CAT (Units/g)	APX (Units/g)	TPC (μM/g)	POD (Units/g)	MDA (μM/g)	TOS (μM/g)	Amylase (mg/g)	TAC (μM/g)	TSS (mg/g)
I	9279.84	628.21	379.59	534.35	12743.13	85.474	177.40	13915.0	90.539	4.46328	41.7817
II	8941.47	632.99	1121.61	823.04	34627.55	621.307	223.16	1838.26	118.357	4.85189	42.7671
III	5661.5	633.37	1383.75	894.99	5606.25	128.837	199.62	1998.25	125.773	2.14198	44.8846

B											
Cluster	RS (mg/g)	NRS (mg/g)	Protease (Units/g)	SOD (Units/g)	Protein (mg/g)	Lycopene (μg/g)	Chl. a (μg/g)	Chl. b (μg/g)	Total chl. (μg/g)	Carotenoids (μg/g)	Total flavonoids (μg/g)
I	12.0005	29.5925	7995.25	198.235	420.416	1.964	50.527	54.459	104.737	3.498	106.936
II	6.76027	36.4470	11699.0	176.792	242.017	6.571	133.894	162.825	294.783	12.277	312.362
III	5.32322	39.1114	11,085	244.754	161.912	4.933	112.263	112.263	212.405	11.306	336.864

### Correlation analysis (Pearson’s test)

Correlation analysis (Pearson’s test) was applied to all biochemical traits for lentil genotypes under investigation at 95% confidence interval. It is depicted that ascorbic acid (AsA) showed a significantly positive correlation with peroxidase (POD) and superoxide dismutase (SOD) and a negative correlation with total soluble proteins (TSP). Additionally, catalase (CAT) is positively correlated with total phenolic content (TPC), malondialdehyde content (MDA), protease (PROT), alpha-amylase (AMYL), lycopene, chlorophyll a, chlorophyll b, total chlorophyll, carotenoids, and total flavonoids (TF) and negatively correlated with total oxidant status (TOS) and total soluble proteins (TSP). Significant positive correlation was found between ascorbate peroxidase (APX), TPC, POD, MDA, protease, alpha-amylase, lycopene, chlorophyll a, chlorophyll b, total chlorophyll, carotenoids, and total flavonoids (TF) and a negative correlation with TOS and TSP. A significant positive association of TPC was noticed between CAT, APX, POD, MDA, NRS, protease, alpha-amylase, lycopene, chlorophyll a, chlorophyll b, total chlorophyll, carotenoids, and total flavonoids (TF) and a negative correlation with TOS, RS, and TSP. POD is positively correlated with AsA, APX, TPC, MDA, protease, alpha-amylase, lycopene, chlorophyll a, chlorophyll b, total chlorophyll, carotenoids, and total flavonoids (TF) and negatively correlated with TOS and TSP. Furthermore, MDA displayed a significant positive correlation with CAT, APX, TPC, POD, protease, alpha-amylase, lycopene, chlorophyll a, chlorophyll b, total chlorophyll, carotenoids, and total flavonoids (TF) and was negatively correlated with TOS, RS, and TSP. In case of TOS, a significant positive association is revealed between RS and TSP but a negative association with CAT, APX, TPC, POD, MDA, NRS, protease, alpha-amylase, lycopene, chlorophyll a, chlorophyll b, total chlorophyll, carotenoids, and total flavonoids (TF). For TS (total soluble sugars), a positive correlation is found between NRS (non-reducing sugars). Reducing sugars (RS) showed a significant positive correlation with TOS and TSP. In case of NRS, a significant positive association was noticed between TPC and TS, while a negative association was found among TOS, RS, and TSP. For protease, a significant positive correlation was detected with CAT, APX, TPC, POD, MDA, alpha-amylase, lycopene, chlorophyll a, chlorophyll b, total chlorophyll, carotenoids, and total flavonoids (TF) and a negative correlation with TOS, RS, and TSP. However, a significant positive correlation was inferred between SOD and AsA but depicted a negative correlation with lycopene, chlorophyll a, chlorophyll b, total chlorophyll, and carotenoids. For TSP, significant positive association was observed with TOS and RS and negative association with AsA, CAT, APX, TPC, POD, MDA, NRS, protease, alpha-amylase, lycopene, chlorophyll a, chlorophyll b, total chlorophyll, carotenoids, and total flavonoids (TF). Next, alpha-amylase showed a significant positive association with CAT, APX, TPC, POD, MDA, protease, lycopene, chlorophyll a, chlorophyll b, total chlorophyll, carotenoids, and total flavonoids (TF) and a negative association with TOS and TSP. The correlation analysis for TAC did not reveal any association with the rest of the studied biochemical parameters. In case of lycopene content, a significant positive association was observed between CAT, APX, TPC, POD, MDA, protease, alpha-amylase, chlorophyll a, chlorophyll b, total chlorophyll, carotenoids, and total flavonoids (TF), and a negative association was noticed with TOS, RS, SOD, and TSP. For chlorophyll a, a significant positive correlation was found between CAT, APX, TPC, POD, MDA, protease, amylase, lycopene, chlorophyll b, total chlorophyll, carotenoids, and total flavonoids (TF), and a negative correlation with TOS, RS, SOD, and TSP. Chlorophyll b showed a significant positive association with CAT, APX, TPC, POD, MDA, protease, amylase, lycopene, chlorophyll a, total chlorophyll, carotenoids, and total flavonoids (TF) and a negative correlation with TOS, RS, SOD, and TSP. In case of total chlorophyll content, a significant positive correlation was observed between CAT, APX, TPC, POD, MDA, protease, alpha-amylase, lycopene, chlorophyll a, chlorophyll b, carotenoids, and total flavonoids (TF). For carotenoids content, a significant positive association was observed between CAT, APX, TPC, POD, MDA, protease, alpha-amylase, lycopene, chlorophyll a, chlorophyll b, total chlorophyll, and total flavonoids (TF), but a significant negative correlation was observed with TOS, RS, SOD, and TSP. Total flavonoids (TF) showed a significant positive association with CAT, APX, TPC, POD, MDA, protease, alpha-amylase, lycopene, chlorophyll a, chlorophyll b, total chlorophyll, and NRS (non-reducing sugars) (Table 6).

**Table 6:** Correlation analysis of the lentil genotypes for studied biochemical traits.

Variables	AsA	CAT	APX	TPC	POD	MDA	TOS	TS	RS	NRS	Protease	SOD	TSP	Amylase	TAC	LYCO	CHLA	CHLB	Total CAR	Total CHLORO	TF
AsA	**1**	0.091	0.140	0.151	**0.228**	0.044	−0.071	0.059	−0.026	0.059	0.070	**0.211**	−**0.214**	0.083	0.036	−0.127	−0.091	−0.136	−0.063	−0.126	0.105
CAT	0.091	**1**	0.154	**0.384**	0.177	**0.352**	−**0.304**	−0.012	−0.087	0.036	**0.395**	−0.004	−**0.324**	**0.251**	−0.151	**0.295**	**0.274**	**0.225**	**0.363**	**0.246**	**0.357**
APX	0.140	0.154	**1**	**0.311**	**0.260**	**0.278**	−**0.266**	−0.002	−0.170	0.085	**0.439**	−0.037	−**0.296**	**0.207**	0.105	**0.333**	**0.325**	**0.293**	**0.359**	**0.301**	**0.431**
TPC	0.151	**0.384**	**0.311**	**1**	**0.541**	**0.502**	−**0.629**	0.052	−**0.271**	**0.204**	**0.648**	−0.161	−**0.532**	**0.365**	0.059	**0.662**	**0.641**	**0.550**	**0.720**	**0.594**	**0.780**
POD	**0.228**	0.177	**0.260**	**0.541**	**1**	**0.342**	−**0.357**	−0.015	−0.163	0.064	**0.385**	−0.065	−**0.214**	**0.209**	0.013	**0.379**	**0.380**	**0.318**	**0.389**	**0.326**	**0.488**
MDA	0.044	**0.352**	**0.278**	**0.502**	**0.342**	**1**	−**0.546**	0.044	−**0.258**	0.179	**0.454**	−0.121	−**0.413**	**0.271**	−0.120	**0.508**	**0.464**	**0.432**	**0.577**	**0.445**	**0.600**
TOS	−0.071	−**0.304**	−**0.266**	−**0.629**	−**0.357**	−**0.546**	**1**	−0.051	**0.297**	−**0.200**	−**0.549**	0.192	**0.548**	−**0.363**	0.137	−**0.600**	−**0.583**	−**0.479**	−**0.666**	−**0.526**	−**0.756**
TS	0.059	−0.012	−0.002	0.052	−0.015	0.044	−0.051	**1**	0.160	**0.848**	−0.093	0.055	−0.069	0.096	0.008	−0.018	−0.019	−0.017	−0.012	−0.006	0.023
RS	−0.026	−0.087	−0.170	−**0.271**	−0.163	−**0.258**	**0.297**	0.160	**1**	−**0.373**	−**0.320**	0.069	**0.234**	−0.133	0.001	−**0.307**	−**0.286**	−**0.250**	−**0.356**	−**0.276**	−**0.369**
NRS	0.059	0.036	0.085	**0.204**	0.064	0.179	−**0.200**	**0.848**	−**0.373**	**1**	0.087	0.001	−**0.198**	0.165	0.018	0.166	0.148	0.138	0.195	0.158	**0.228**
Protease	0.070	**0.395**	**0.439**	**0.648**	**0.385**	**0.454**	−**0.549**	−0.093	−**0.320**	0.087	**1**	−0.150	−**0.540**	**0.469**	0.045	**0.665**	**0.657**	**0.569**	**0.713**	**0.617**	**0.754**
SOD	**0.211**	−0.004	−0.037	−0.161	−0.065	−0.121	0.192	0.055	0.069	0.001	−0.150	**1**	−0.035	−0.041	−0.009	−**0.411**	−**0.366**	−**0.420**	−**0.355**	−**0.408**	−0.132
TSP	−**0.214**	−**0.324**	−**0.296**	−**0.532**	−**0.214**	−**0.413**	**0.548**	−0.069	**0.234**	−**0.198**	−**0.540**	−0.035	**1**	−**0.330**	0.009	−**0.518**	−**0.524**	−**0.418**	−**0.582**	−**0.463**	−**0.715**
Amylase	0.083	**0.251**	**0.207**	**0.365**	**0.209**	**0.271**	−**0.363**	0.096	−0.133	0.165	**0.469**	−0.041	−**0.330**	**1**	0.014	**0.366**	**0.324**	**0.347**	**0.367**	**0.338**	**0.457**
TAC	0.036	−0.151	0.105	0.059	0.013	−0.120	0.137	0.008	0.001	0.018	0.045	−0.009	0.009	0.014	**1**	−0.007	0.063	0.038	−0.014	0.046	0.048
LYCO	−0.127	**0.295**	**0.333**	**0.662**	**0.379**	**0.508**	−**0.600**	−0.018	−**0.307**	0.166	**0.665**	−**0.411**	−**0.518**	**0.366**	−0.007	**1**	**0.967**	**0.962**	**0.969**	**0.975**	**0.732**
CHLA	−0.091	**0.274**	**0.325**	**0.641**	**0.380**	**0.464**	−**0.583**	−0.019	−**0.286**	0.148	**0.657**	−**0.366**	−**0.524**	**0.324**	0.063	**0.967**	**1**	**0.943**	**0.941**	**0.976**	**0.716**
CHLB	−0.136	**0.225**	**0.293**	**0.550**	**0.318**	**0.432**	−**0.479**	−0.017	−**0.250**	0.138	**0.569**	−**0.420**	−**0.418**	**0.347**	0.038	**0.962**	**0.943**	**1**	**0.889**	**0.988**	**0.592**
TOTAL CAR	−0.063	**0.363**	**0.359**	**0.720**	**0.389**	**0.577**	−**0.666**	−0.012	−**0.356**	0.195	**0.713**	−**0.355**	−**0.582**	**0.367**	−0.014	**0.969**	**0.941**	**0.889**	**1**	**0.923**	**0.808**
TOTAL CHLORO	−0.126	**0.246**	**0.301**	**0.594**	**0.326**	**0.445**	−**0.526**	−0.006	−**0.276**	0.158	**0.617**	−**0.408**	−**0.463**	**0.338**	0.046	**0.975**	**0.976**	**0.988**	**0.923**	**1**	**0.646**
TF	0.105	**0.357**	**0.431**	**0.780**	**0.488**	**0.600**	−**0.756**	0.023	−**0.369**	**0.228**	**0.754**	−0.132	−**0.715**	**0.457**	0.048	**0.732**	**0.716**	**0.592**	**0.808**	**0.646**	**1**

Values in bold are different from 0 with a significance level of alpha = 0.05. AsA = ascorbic acid, CAT = catalase, APX = ascorbate peroxidase, POD = peroxidase, MDA = malondialdehyde, TOS = total oxidant status, TS = total soluble sugars, RS = reducing sugars, NRS = non-reducing sugars, SOD = superoxide dismutase, TSP = total soluble proteins, Amylase = alpha-amylase, TAC = total antioxidant capacity, LYCO = lycopene, CHLA = chlorophyll a, CHLB = chlorophyll b, TOTAL CAR = total carotenoids, TOTAL CHLORO = total chlorophyll.

## Discussion

Due to the excessive formation of cellular reactive oxygen species (ROS), the cascade of reactions occurring in the extracellular matrix and tissues causes the activation of the endogenous defense mechanisms. As a result of these defense mechanisms (enzymatic or non-enzymatic), inactivation of ROS takes place (43, 44). To explore the comprehensive nutritional and antioxidant potential of the lentil seeds, various biochemical analyses were conducted on the lentil seed flour in the current study.

Ascorbic acid (vitamin C) reacts directly with hydroxyl radicals and superoxide (an ion radical) in wheat and other crops (45). It helps in reducing the effects of lipid peroxidation (46). Ascorbic acid plays an important role as a plant growth regulator *via* the hormone signaling pathway. It also acts as a modulator in several physiological processes, i.e., flowering, membrane permeability, seed germination, photosynthesis, transport of ions from roots to the whole plant, respiration, and senescence (47). As germination time increases, ascorbic acid content in lentil sprouts increases, with no significant effect on temperature. In the current study, only five genotypes were placed in high class. The highest content of ascorbic acid was found in LPP 12182 (706 μg/g s. wt.), which demonstrates similar results as found in wheat genotype SH-2002 (713 μg/g s. wt.) in the previous study (45). Twenty-eight genotypes were categorized into medium class. In another study, mung bean seeds exhibited 490 μg/g s. wt. ascorbic acid content, which is slightly higher than the ascorbic acid content (333 μg/g s. wt.) observed in the present study (48).

In addition to carbohydrates, dietary fibers, and proteins, lentils contain many other bioactive compounds like peptides and phenolic compounds, which have several health benefits (21). Phenolic compounds are important components of lentils as well as an essential source of antioxidant compounds (20). Among the major non-enzymatic antioxidants, total phenolic compounds and total flavonoids possess the major bioactive compounds. They play crucial roles in the functional and structural roles of our body and are also constituents of parts of plants (49, 50). Among the tested genotypes, only 14 were included in the intermediate TF class, and 52 genotypes possessed low TF values. The highest TF value was observed in NLI 17059 (366.42 μg/mL sample), which is relatively similar to that reported in chickpea desi wild hybrid WH-1 (394 μg/mL sample) (51). Another study explained the highest TF values in lentil cultivars ranging from 0.96 to 1.93 mg CAE/g (52), which is relatively higher compared to the values detected in lentil genotypes ranging from 300.01 to 367 μg/mL sample in the current study. The genotypes with the highest TFs can be utilized by breeders to ameliorate the availability of micronutrients in various nutraceuticals.

For TPC values, 31 genotypes were grouped as high class, 14 as intermediate class, and 55 as low class. Generally, the highest TPC value was observed in NLI 17003 (54,600 μM/g s. wt.), which is higher than reported in the approved chickpea variety CM-98 (desi type) (34,725 μM/g s. wt.) (51). In another study, the TPC value noticed in lentil genotype IG129294 was 4.50 mg GAE/g (53) which is higher as compared to the TPC value depicted in the current study, LHM 17010 (4,755 μM/g sample).

The digestibility and biological utilization of legumes are adversely affected by antinutritional compounds such as phytates, tannins, and trypsin inhibitors (54). Tannins are hydrolyzable compounds that comprise glucose molecules condensed with simple phenolic chains like gallic acid. In a previous study, the lowest tannin value (0.54 mg/g) was detected in genotype NDL-1 (55) which is even higher than the tannin content found in LPP 11137 (257 μM/g s. wt.). In addition, lentil contains high flavonoids, condensed tannins, and phenolic contents (1.30, 5.97 mg catechin equivalents g^−1^, and 6.56 mg gallic acid equivalents g^−1^), respectively (56, 57) which is comparatively higher than the highest tannin value found in NLI 17057 (24,563 μM/g s. wt.) elaborated in the current study. In the context of the current findings, it is depicted that genotypes with low tannin values can be used for the development of genotypes with enhanced mineral bioavailability and better nutritional attributes.

Catalase is the most crucial enzyme that neutralizes hydrogen peroxide (H_2_O_2_) by using oxygen and water in the presence of magnesium and iron as cofactors. It has the highest K_cat_ value among all antioxidant enzymes (58). Catalase plays a crucial role in the oxidative reaction, occurring during bleaching and bread making, and prevents the harmful effects of H_2_O_2_ (59). Due to environmental stress, an oxidative stress condition occurs in plants, which is mitigated by the expression of certain catalase isozymes (60). In the present study, high levels of catalase ranged from 2001 to 5,600 Units/g s. wt., and 10 genotypes were found in this group. The highest value of catalase was found in LPP 12110 (5,595 Units/g s. wt.), which is comparatively higher than the amount of catalase in chickpea genotype ICC-4951 (893 ± 50 Units/g s. wt.) evaluated in other case studies (51). Genotypes were classified in the medium class, ranging from 1,001 to 2000 Units/g s. wt. which shares a similar range of catalase activity (15,240 Units/g s. wt.) found in black gram (61). As a result of the present study, three approved varieties, including Masoor 93, Shiraz-96, and Punjab Masoor 2009, exhibited a medium range of catalase activity. Furthermore, one exotic approved variety, Lentil black, was among the varieties with a higher level of catalase activity.

Ascorbate peroxidase (APX) plays a crucial role in the glutathione peroxidase cycle and is also used for the removal of hydrogen peroxide. It also performs an important function for plant cell protection from certain environmental stresses (62). It was noticed in the current study that high APX levels were found in 14 genotypes, ranging from 1,001 to 2,500 Units/g s. wt. Among the tested genotypes, the highest activity was found in ALTINOPARK (2,465 Units/g s. wt.), but the highest APX activity in the wheat genotype was observed in Pavon (1426.67 Units/g s. wt.) in the previous study (45), which is notably lower than lentils. The medium class included 26 genotypes ranging from 701 to 1,000 Units/g s. wt. Low levels were found in 60 genotypes, including ICARDA lines (ILL 6002, ILL 2245, ILL 2580, ILL 4400, ILL 8006, ILL 7978, X 2013–174-1, X 2011S-160-22, X 2011S 33–34-32, X 2011S-19-12, X 2011S-19-39), ranging ≤700 Units/g s. wt., and the lowest activity was observed in X-2013-174-1 and NLM 15011 (41.500 Units/g s. wt.).

Peroxidase (POD) helps in scavenging (ROS) reactive oxygen species, which results in oxidative cell damage (63). Several plant peroxidases have been recognized as biochemical markers for various biotic and abiotic types of stresses because they play essential roles in crucial physiological processes such as lignification of the cell wall, auxin catabolism, and cross-linkage of structural proteins and pectins in the cell wall (60). High levels of POD were found in six genotypes ranging from 1,501 to 3,200 Units/g s. wt., and the highest activity was shown by NIAB Masoor 2002 (3,170 Units/g s. wt.). LPP 12062 (63 Units/g s. wt.). Genotypes ranging ≤500 Units/g s. wt. were placed under a low category, and 82 genotypes were included in this group. Moreover, the local approved variety Shiraz-96 exhibited (142 Units/g) POD activity, which shares a similar range value with the lentil variety (154 μmol min^−1^ g^−1^) described in the previous study (48).

Superoxide dismutase (SOD) is important in preventing the spread of unstoppable free radicals and aids in the antioxidant activity of peroxidase and catalase. Elevated levels of POD, SOD, and CAT were observed during water stress conditions, which help in scavenging free radicals (64). In the current study, the highest level of SOD was found in LPP 11195 (294.096 Units/g s. wt.) as well as the highest SOD value elaborated in the wheat variety Manthar-2003 (278.93 Units/g s. wt.). In the present study, the lowest SOD activity was observed in LHM 17019 (66.97 Units/g s. wt.), which depicted nearly similar results (76.17 Units/g s. wt.) in the wheat variety Punjab-2011 (45).

Proteolytic enzymes show remarkable performance in developing and maintaining the physiology of plants and are known as a source of amino acids for various novel protein synthesis processes (65). Microorganisms are the key contributors to protease production, and plant-based proteases have not been well studied so far (66). Previously conducted studies reveal the occurrence of proteolytic enzymes in various beans and plant seeds, e.g., sorghum (67), potato tubers (68), barley (69), mung bean (70), rice (71), and moringa olifera seeds (72). In this study, Masoor 93 exhibited the highest protease value (16,050 Units/g. s. wt.). The lowest value was noticed in LPP 11111 (6,125 Units/g. s. wt.), which is comparatively higher as compared to the protease activity noticed in wheat seeds (1,000 Units/g sample) (73). Similarly, protease activity in the maize variety Golden Silks (22,488 Units/g sample) (74) was noteworthy higher than that evaluated in the approved lentil variety Masoor 93 (16,050 Units/g s. wt.) in the current study.

Hydrolytic enzymes, such as proteases, alpha-amylase, and esterase, specifically split larger biomolecules into smaller ones *via* hydrolysis. In this process, the addition of one or more water molecules takes place (75). Alpha-amylase is a hydrolyzing enzyme that catalyzes the hydrolysis of 4-glycosidic linkages in starch to yield products such as glucose and maltose. These products can be extracted from animals, plants, and microorganisms (76). Alpha-amylase plays an essential role in absorption mechanisms like the gassing ability of dough and in maintaining the properties of bread (77). In lentil seeds, amylases play an important role in providing utilizable substrates for growth and development (78). In this study, high levels of alpha-amylase were found in four genotypes ranging from 151 to 273 mg/g. s. wt., and the highest value was found in LPP 12137 (271.87 mg/g. s. wt.), which is comparatively higher than alpha-amylase activity in chickpea (kabuli advance line), i.e., CH74/08 (213.02 ± 3.20 mg/g s. wt.) (51). However, 54 genotypes were grouped at an intermediate level, ranging from 101 to 150 mg/g s. wt. LPP 12068 (112 mg/g s. wt.) showed similar alpha-amylase activity as reported in previously reported wheat seeds (112 mg/g sample) (79).

Malondialdehyde (MDA) is produced as a result of alterations in cell membrane characteristics like disability of enzymatic function and oxidative degradation of cell components (80). MDA level also shows the extent of damage to the plant cell (62). Lipid peroxidation is the major reason for seed injury for storage purposes and is also responsible for the initial biochemical changes in the seed for storage. Increased MDA content is due to accelerating aging and prolonged storage (81). In this study, high levels of MDA were noticed in 24 genotypes, ranging from 231 to 301 Units/g s. wt. Physiological analysis of different wheat genotypes revealed that the MDA content in wheat flour is much higher than in lentil flour. The highest level of MDA was noticed in wheat genotype Margalla-99 (679.23 μM/g s. wt.) (45) and lupine (*Lupinus termis*) seeds (380 μM/g sample) (82) which is much higher than the approved variety Punjab Masoor 2009 (300.5 μM/g s. wt.).

In essence, total oxidant status (TOS) in living organisms is referred to as the gross oxidation status of the body (83). Antioxidant capacity of a body can be determined by evaluating its total oxidant status (84). Oxidative stress is the consequence of the imbalance between the body’s immune system mechanisms and reactive oxygen species (ROS), resulting in cellular damage (85). Pulses revealed a significant relationship between antioxidant capacity and polyphenols (86, 87). Additionally, lentil is considered a beneficial and nutrient-rich food as it contains essential nutrients and phytochemicals (87). In the present study, it was observed that high levels of TOS were observed in 17 genotypes ranging from 15,001 to 48,000 μM/g. s. wt., and the highest TOS value was recorded in NLM 15029 (47,487 μM/g. s. wt.), which is much higher compared to the highest TOS value reported in CM3457/91 (356 ± 17.50 μM/g s. wt.) (51). The above-discussed results showed that the oxidation status in the studied lentil varieties is far higher than that noticed in chickpeas.

An increased interest has been developed for food security reasons and environmental stability in search of new sources of proteins as an alternative source to avoid animal proteins. Pulse proteins, especially lentil proteins, exhibit a wide range of functional properties (88). With enzymatic and thermal modifications, the nutritional attributes of lentil proteins are further improved (89). In another study, there was a considerable higher protein content in faba bean (330 mg/g s. wt.) than in red lentil (285 mg/g s. wt.), followed by yellow pea (257 mg/g s. wt.) (90). Like other legumes, lentil is a protein-rich crop with a protein content of 20.4–31.4% (91). In the present study, significant variation was observed among the tested lentil genotypes. Highest protein content was found in X 2011S-19-39 (546.33 mg/g s. wt.), which is comparatively lower than found in red lentil (285 mg/g s. wt.) (90). Another previous study demonstrated (314 mg/g s. wt. sample) protein content in lentil seed flour (92) which shows a similar range as depicted in NL 96635 (311.5 mg/g s. wt.) in the current investigation.

Total soluble sugar is an essential physiological parameter that plays a significant role in seed development and production (93–95). During germination, the stored insoluble nutrients present in the cotyledon are converted to soluble ones through the hydrolysis of any macromolecule (96). Moreover, total soluble sugars enhance stress tolerance in seeds with improved storability during stress conditions (97). In a previous study, total, non-reducing, and reducing sugar content in lentil seeds were 50.1, 34.9, and 15 mg/g s. wt., respectively (98). As a result of the current study, 20 lentil genotypes exhibited higher levels of total soluble sugars ranging from 51 to 85 mg/g. s. wt., and NLM 15015 (83.9 mg/g s. wt.) showed the highest value. Low levels were found in 16 genotypes, but the lowest value was noticed in GAEIL (11.49 mg/g. s. wt.). The previously reported total soluble sugar content in lentil seeds ranged from 34.5 to 70.5 mg/g s. wt. In a similar study, the lentil genotype LL-1277 (70 mg/g s. wt.) exhibited the highest TSS value (2) which is slightly lower than the TSS value observed in NLM 15015 (83.9 mg/g s. wt.) in the current study. Conversely, lower TSS content was observed in a cow pea genotype PI201498 (70.1 mg/g s. wt.) (99) than determined in NLM 15015 (83.9 mg/g s. wt.) in the present study. Pea *(Pisum sativum)* seeds demonstrated higher reducing and non-reducing sugar contents (100) compared to the values obtained in the current investigation.

Legume foods have gained utmost importance due to the presence of various micronutrients, macronutrients, and surplus amounts of phytochemicals. These phytochemicals exhibit various health-promoting effects for the prevention of chronic diseases (101, 102). Additionally, this positive health impact is caused by high antioxidant content of legumes (103). The production of free radicals is responsible for the destruction of micronutrients, DNA, carbohydrates, proteins, and unsaturated lipids (102). The pathogenicity caused by free radical production can be minimized by utilizing antioxidants from plants and plant-based products (104).

In comparison with other legumes, lentils exhibit an ample amount of phenolic compounds that are well known to have high antioxidant capacity (20, 21). In lentil, high antioxidant capacity (TAC) is attributed to the existence of several phenolic (bound and free) compounds. Moreover, extractable phytochemicals showed less antioxidant activity as compared to the bound phytochemicals in case of lentil (105). In another study, a higher total antioxidant capacity in lentil seeds was reported than in pea seeds. It has been reported in earlier studies that lentil consumption plays an important role in reduction of various diseases, such as diabetes, cancer, obesity, and other cardiovascular diseases, due to its polyphenolic properties, which are responsible for its anti-inflammatory, antioxidant, and nephroprotective properties (106). For instance, flavonoids in lentil have been shown to control alpha-lipases and glucosidases, resulting in maintaining blood glucose levels (107). The oxygen radical absorbing capacity (ORAC) in lentils (8.43 μM/g. s. wt.) was comparatively higher than yellow pea (1.17 μM/g. s. wt.) and chickpea (1.04 μM/g. s. wt.) (108). In the current investigation, lentil genotype NLM 15016 (15.76 μM/g. s. wt.) possessed similar TAC values as determined in lentil seeds (14 μM/g. s. wt.) in the previous study (109). In addition to a similar study, low TAC was noticed in pea seeds (1.9 μM/g. s. wt.) as compared to lentil seeds (109).

During germination, chlorophyll content is not completely deteriorated in ripening seeds, but appreciable quantities can be determined in mature seeds (110). The existence of chlorophyll in seeds was first demonstrated by Monteverde and Lubimenko in 1909 (111). Furthermore, seed quality is the measure of chlorophyll content present in it, and both are inversely related to each other. Hence, the seeds with increased chlorophyll content have a declining germination rate (112).

All types of seeds are prone to the process of aging as well as quality deterioration, which eventually leads to the loss of seed viability (113).

The green color of the plant and seed is due to the presence of a pigment called chlorophyll (114). There are two types of chlorophyll, such as chlorophyll a and b, present in green algae and terrestrial plants. Chlorophyll a exhibits a green-blue color, and chlorophyll b contains a green-yellow color (39). In the present study, the least (chlorophyll a and chlorophyll b) values were noticed in LHM 17010 (25.14 μg/g s. wt.) and NLH 11211 (8.39 μg/g s. wt.), respectively, which are higher than the values for chlorophyll a (11.83 μg/g s. wt.) and chlorophyll b (3.01 μg/g s. wt.) reported in soya bean seeds (115). Low and medium (chlorophyll a) content was noticed in 57 and 40 lentil genotypes, while two approved varieties (NIAB Masoor 2002, Turk Masoor) were among the high class, along with one local hybrid (NLH 12097). On the other hand, NLH 17010 possessed the least chlorophyll a content, and NLH 11211 possessed the least chlorophyll b and total chlorophyll content. Overall, NLH 12097 showed highest chlorophyll a, chlorophyll b, and total chlorophyll contents among all tested genotypes.

In addition, carotenoids are essential pigments present in many parts of plant organs and tissues and are also found in the seeds of higher plants. They have a key role as antioxidants and prevent the seeds from generating reactive oxygen species (ROS) from the process of aging (116, 117). Comparatively low, medium, and high levels of carotenoids were found in 44, 22, and 34 genotypes, respectively. Markaz 2009 was the approved variety with the highest carotenoid content. Moreover, in the current investigation, the least carotenoid content was found in ICARDA line ILL 6002 (1.31 mg/g s. wt.), which is slightly higher than found in lentil seeds (0.45 mg/g s. wt.) (118) in the previous study.

In case of lycopene content based on various studied attributes in this study, significant variation was observed. In total, 67 and 30 genotypes were included in low and intermediate classes, respectively. However, only three genotypes were assorted in high class, including one exotic variety (Turk Masoor), one local variety (Markaz 2009), and one local hybrid (NLH 12097).

Among all tested genotypes, NLH 12097 (10.881 μg/g s. wt.) showed the highest lycopene content and NLH 11211 (0.686 μg/g s. wt.) exhibited the least value as observed in case of chlorophyll as noticed in case of (chl. a, b and total chlorophyll). Furthermore, in recent studies, the highest lycopene content was present in chickpea (desi type) Sheenghar-2000 (12.579 μg/g s. wt.) (51), which is relatively higher as compared to NLH 12097 (10.881 μg/g sample) in this study.

Correlation is the study of an association between different variables. In correlation analysis, the variation in magnitude of one variable is directly linked with the change in magnitude of the other variable (either positive or negative correlation) (119). In the current investigation, it was depicted that CAT, APX, TPC, POD, and MDA showed similar trends in terms of significant positive and negative correlation. Similar negative correlation trend was observed for TOS, RS, SOD, and TSP with pigments in this study. Total antioxidant capacity (TAC) is not significantly correlated, either positively or negatively, with other studied biochemical attributes of the genotypes under the present study. Furthermore, it is noticed that lycopene, chlorophyll a, b, total chlorophyll, and total carotenoids, except total flavonoids (TF), followed similar trends in terms of positive and negative associations. In another study, a significant positive correlation was reported between total antioxidant activity and total flavonoids (120, 121) which is not similar to the current findings. The reason behind this difference is that antioxidant activity is not the result of one or more phytochemicals but is an interactive and synergistic effect of various bioactive compounds found within a plant at a particular time and stage (122).

Principal component analysis (PCA) is a multivariate technique, which is applied to simplify large data sets and to derive useful information from the analyzed data (123). In the current study, five principal components were responsible for 69.23% of the total variation. The most differential parameters in PC-I with positive vector loadings were AsA, CAT, APX, TPC, POD, MDA, TSS, NRS, protease, alpha-amylase, TAC, lycopene, chlorophyll a, b, total chlorophyll, carotenoids, and total flavonoids. Among all recognized parameters, a single variable is mostly selected (124). High factor loadings were noticed in carotenoids and lycopene, with values of 0.324 and 0.316, respectively. It is depicted that total carotenoids exhibited the best single-loading factor. Regarding PC-II, 12 traits showed positive factor loadings such as AsA, CAT, APX, TPC, POD, MDA, TSS, NRS, protease, SOD, alpha-amylase, and total flavonoids, while NRS exhibited the highest positive factor loading value (0.439), followed by TSS (0.424). The distance between the biplot origin and genotypes illustrates the genotypic variations from the total mean, so the short and long distances of all genotypes from the origin can be utilized to evaluate the poorest and excellent genotypes (125). The current findings revealed that the approved varieties, including Punjab Masoor 2009, NIAB Masoor 2002, Lentil Black, Turk Masoor, Shiraz-96, Punjab Masoor 2019, and one local genotype, NLH 12097, were spotted distant from the origin and hence declared as the best performers among the concerning genotypes regarding all traits in this investigation. The genotypes found near the biplot origin were bad performers compared to the distant ones.

Clustering is also a multivariate statistical technique, which combines diverse genotypes/traits into two subsequent groups on the basis of resemblance in their expression (126). Here, we concluded that various lentil genotypes showed significant divergence among different biochemical and nutritional attributes. Cluster I consisted of genotypes with higher mean values for tannins, reducing sugars (RS), and protein content, while cluster III comprised genotypes with maximum AsA, CAT, APX, alpha-amylase, TSS, NRS, and SOD mean values. Furthermore, it was observed that cluster II genotypes showed remarkable divergence with cluster III, as shown in Table 4. It is evident from the current findings that cluster analysis is considered one of the most important tools for the classification of germplasm. It also provides a consistent foundation for the classification of material to prepare breeding tactics for upcoming scientific research (127, 128). The subsequent results from the present study revealed that multivariate analysis helps in assembling genotypes into various clusters on the basis of their respective PCs.

## Conclusion

The current findings revealed that local, exotic lentil genotypes and approved varieties have noteworthy potential for the studied nutritional and biochemical parameters. Most of the ICARDA lines used in the current study are proven to be the better source of plant-based proteins. Moreover, lentil genotypes, such as NLI 17003, NLI 17059, and NLM 15016, possess high total antioxidant capacities, which can be utilized by breeders to improve the antioxidant potential and nutraceutical value of lentil and lentil-based products. Additionally, the X 2011S 33–34-32 genotype can be used by the food industry in making pasta, multigrain bread, and snacking foods due to its high protein content for meat alternative seekers. One of the locally approved varieties, Markaz-2009, is considered to have the highest total carotenoid content among the studied genotypes. Lentil can be incorporated into staple foods due to its ability to provide nutraceutical and functional benefits to millions of consumers worldwide. The identified genotypes with high nutritional values can be utilized for improvement in the nutritional aspects of lentils.

## Data availability statement

The original contributions presented in the study are included in the article/Supplementary material, further inquiries can be directed to the corresponding author.

## Author contributions

FR: Data curation, Formal analysis, Methodology, Writing – original draft. AH: Conceptualization, Funding acquisition, Project administration, Resources, Supervision, Writing – review & editing. MA: Resources, Writing – review & editing.
