# Supplementary material for: Grain nutritional and antioxidant profiling of diverse lentil (Lens culinaris Medikus) genetic resources revealed genotypes with high nutritional value
Source: Front Nutr. 2024 Mar 22;11:1344986. doi: 10.3389/fnut.2024.1344986 (PMC10998453; doi:10.3389/fnut.2024.1344986)
Supplement: Supplementary file 1 [file Table_1.DOCX]

**Figure S1** shows means and standard error bars for Ascorbic acid (AsA) [A] and Total phenolic content (TPC) [B] in seeds of different lentil genotypes. Means and alphabets are significantly different with (Tukey’s HSD p˂0.05).

**Figure S2** shows means and standard error bars for tannins [A] and catalase [B] in seeds of different lentil genotypes. Means and alphabets are significantly different with (Tukey’s HSD p˂0.05).

**Figure S3:** shows means and standard error bars for ascorbate peroxidase (APX) [A] and peroxidase [B] in seeds of different lentil genotypes. Means and alphabets are significantly different with (Tukey’s HSD p˂0.05).

**Figure S4**  shows means and standard error bars for superoxide dismutase(SOD) [A] and α-Amylase [B] in seeds of different lentil genotypes. Means and alphabets are significantly different with (Tukey’s HSD p˂0.05).

**Figure S5** shows means and standard error bars for protease [A] and Total oxidant status (TOS) [B] in seeds of different lentil genotypes. Means and alphabets are significantly different with (Tukey’s HSD p˂0.05).

**Figure S6** shows means and standard error bars for malondialdehyde content (MDA) in seeds of different lentil genotypes. Means and alphabets are significantly different with (Tukey’s HSD p˂0.05).

**Figure S7** shows means and standard error bars for protein content [A] and total soluble sugars (TSS) [B in seeds of different lentil genotypes. Means and alphabets are significantly different with (Tukey’s HSD p˂0.05).

**Figure S8** shows means and standard error bars for reducing sugars (RS) [A] and non-reducing sugars (NRS) [B] in seeds of different lentil genotypes. Means and alphabets are significantly different with (Tukey’s HSD p˂0.05).

**Figure S9** shows means and standard error bars for total antioxidant capacity (TAC) [A] and total flavonoids (TF) [B] in seeds of different lentil genotypes. Means and alphabets are significantly different with (Tukey’s HSD p˂0.05).

**Figure S10** shows means and standard error bars for carotenoids [A] and chlorophyll a [B] in seeds of different lentil genotypes. Means and alphabets are significantly different with (Tukey’s HSD p˂0.05).

**Figure S11** shows means and standard error bars for chlorophyll b [A] and total chlorophyll [B] in seeds of different lentil genotypes. Means and alphabets are significantly different with (Tukey’s HSD p˂0.05).

**Figure S12:** shows means and standard error bars for lycopene content in seeds of different lentil genotypes. Means and alphabets are significantly different with (Tukey’s HSD p˂0.05).

**Table S1.** Categorization of lentil germplasm in low, medium and high levels based on respective values for studied biochemical attributes.

| Sr.# | Parameters | | Low | Genotypes | Medium | Genotypes | High | Genotypes |  |  | |  | | |
| --- | --- | --- | --- | --- | --- | --- | --- | --- | --- | --- | --- | --- | --- | --- |
| 1. | Tannins | ≤ 5000 | | NLM 15012, NLM 15014, NLM 15016, LPP 11025, LPP 11137, LPP 12068, LPP 12103, LPP 12105, LPP 12182, NLI 17058, NLI 17059, LHM 17009, Masoor 85, NIA Masoor 05 | 5001-9999 | NLM 15011, NLM 15018, NLM 15020, NLM 15025, NLM 15029, LPP 11001, LPP 12137, NLH 12088, NLH 12096, NLH 11229, NLH 12159, NLH 12196, NLH 15032, NLH 17026, LHM 17006, LHM 17008, LHM 17010, LHM 17013, LHM 17020, LHM 17022, LHM 17023, LHM 17024, Mansehra- 89, Masoor 93, Shiraz- 96, Chakwal Masoor, Punjab Masoor 2019, Punjab Masoor 2020, NL 96635, NL 96700, Turk Masoor, Lentil Black, TCL 85-1, ALTINOPARK, SEYRAN-96, GAEIL, ILL 18108, Precoz, ILL 6002, ILL 2580, ILL 8006, ILL 7978, X 2013-174-1, X 2011S-19-39, | 10000-25000 | NLM 15015, NLM 15019, NLM 15021, NLM 15026, NLM 15035, LPP 11111, LPP 11145, LPP 11168, LPP 11195, LPP 11224, LPP 12051, LPP 12052, LPP 12062, LPP 12110, NLH 11211, NLH 11220, NLH 12097, NLH 12187, NLH 15003, NLH 17034, NLH 17039, NLI 15044, NLI 17001, NLI 17002, NLI 17003, NLI 17057, LHM 17011, LHM 17014, LHM 17015, LHM 17019, LHM 17021, NIAB Masoor 2006, Markaz 2009, Punjab Masoor 2009, NL 96621, NL 96680, ILL 2245, ILL 4400, X 2011S-160-22, X 2011S 33-34-32, X 2011S-19-12, | | |  | | |  |
| 2. | TPC | ≤15000 | | NLM 15011, NLM 15012, NLM 15015, NLM 15016, NLM 15018, NLM 15019, NLM 15020, NLM 15021, NLM 15029, NLM 15035, LPP 11111, LPP 11137, LPP 11145, LPP 11168, LPP 11195,LPP 11224, LPP 12051, LPP 12052, LPP 12062, LPP 12068, LPP 12103, NLH 12088, NLH 11211, NLH 11220, NLH 11229, NLH 12159, NLH 12187, NLH 15032, NLH 17026, LHM 17006, LHM 17010, LHM 17011, LHM 17013, LHM 17014,LHM 17015, LHM 17019, LHM 17020, LHM 17021, LHM 17022, LHM 17023, LHM 17024, Punjab Masoor 2020, ALTINOPARK, SEYRAN-96, GAEIL, ILL 6002, ILL 2245, ILL 2580, ILL 4400, ILL 8006, ILL 7978, X 2013-174-1, X 2011S-160-22, X 2011S 33-34-32, X 2011S-19-39, | 15001-30000 | NLM 15026, LPP 11025, LPP 12105, NLI 17058, NLI 17059, Chakwal Masoor, Punjab Masoor 2019, NL 96621, NL 96680, NL 96700, Turk Masoor, TCL 85-1, | 30001-54600 | NLM 15014, NLM 15025, LPP 11001, LPP 12110, LPP 12137, LPP 12182, NLH 12096, NLH 12097, NLH 15003, NLH 17034, NLH 17039, NLI 15044, NLI 17001, NLI 17002, NLI 17003, NLI 17057, LHM 17008, LHM 17009, Mansehra- 89, Masoor 93, Shiraz- 96, NIAB Masoor 2002, NIA Masoor 05, NIAB Masoor 2006, Markaz 2009, Punjab Masoor 2009, NL 96635, Lentil Black, ILL 18108, Precoz, X 2011S-19-12, | | | | |  |  |
| 3. | AsA | ≤640 | | NLM 15011, NLM 15012, NLM 15014, NLM 15015, NLM 15016, NLM 15018, NLM 15020, NLM 15021, NLM 15029, LPP 11001, LPP 11025, LPP 11111, LPP 11137, LPP 11145, LPP 11195, LPP 12051, LPP 12052, LPP 12068, LPP 12103, NLH 12088, NLH 11211, NLH 11229, NLH 12097, NLH 12159, NLH 12187, NLH 15003, NLH 15032, NLH 17034, NLI 17002, NLI 17058, NLI 17059, LHM 17006, LHM 17009, LHM 17011, LHM 17014, LHM 17019, LHM 17020, LHM 17021, LHM 17023, LHM 17024, Masoor 85, Masoor 93, Shiraz- 96, NIAB Masoor 2002, NIA Masoor 05, NIAB Masoor 2006, Markaz 2009, Chakwal Masoor, Punjab Masoor 2019, Punjab Masoor 2020, NL 96621, NL 96635, NL 96680, NL 96700, Turk Masoor, Lentil Black, ALTINOPARK, GAEIL, ILL 18108, Precoz, ILL 2580, ILL 4400, ILL 8006, ILL 7978, X 2011S-160-22, X 2011S-19-12, X 2011S-19-39, | 641-680 | NLM 15019,NLM 15026, NLM 15035, LPP 11168, LPP 11224, LPP 12062, LPP 12110, LPP 12137, NLH 12096, NLH 11220, NLH 12196, NLH 17026, NLH 17039, NLI 15044, NLI 17003, LHM 17008, LHM 17010, LHM 17013, LHM 17015, LHM 17022, Mansehra- 89, Punjab Masoor 2009, TCL 85-1, SEYRAN-96, ILL 6002,ILL 2245, X 2013-174-1, X 2011S 33-34-32, | 681-706 | NLM 15025, LPP 12105, LPP 12182, NLI 17001, NLI 17057 |  |  | |  | | |
| 4. | CAT | ≤1000 | | NLM 15011, NLM 15012, NLM 15014, NLM 15015, NLM 15016, NLM 15018, NLM 15020, NLM 15021,NLM 15025, NLM 15026, NLM 15029, NLM 15035, LPP 11025, LPP 11111, LPP 11137, LPP 11145, LPP 11168, LPP 11195, LPP 11224, LPP 12051, LPP 12052, LPP 12062, LPP 12103, LPP 12182, NLH 12088, NLH 12096, NLH 11211, NLH 11220, NLH 11229, NLH 12196, NLH 15032, NLH 17026, NLH 17034, NLI 17001, NLI 17003, NLI 17057, NLI 17058, NLI 17059, LHM 17006, LHM 17009, LHM 17010, LHM 17011, LHM 17013, LHM 17014, LHM 17015, LHM 17019, LHM 17020, LHM 17021, LHM 17022, LHM 17023, LHM 17024, Masoor 85, Mansehra- 89, NIAB Masoor 2002, NIA Masoor 05, NIAB Masoor 2006, Markaz 2009, Chakwal Masoor, Punjab Masoor 2019, Punjab Masoor 2020, NL 96621, NL 96680, NL 96700, ALTINOPARK, SEYRAN-96, GAEIL, ILL 18108, Precoz, ILL 6002, ILL 2245, ILL 2580, ILL 4400, ILL 8006, ILL 7978, X 2013-174-1, X 2011S-160-22, X 2011S-19-12, X 2011S-19-39 | 1001-2000 | LPP 11001, NLH 12097, NLH 17039, NLI 17002, LHM 17008, Masoor 93, Shiraz- 96, Punjab Masoor 2009, NL 96635, Turk Masoor, X 2011S 33-34-32, | 2001-5600 | LPP 12068, LPP 12105, LPP 12110, LPP 12137, NLH 12159, NLH 12187, NLH 15003, NLI 15044, Lentil Black, TCL 85-1 |  |  | |  | | |
| 5. | APX | ≤700 | | NLM 15011, NLM 15012, NLM 15015, NLM 15016, NLM 15018, NLM 15019, NLM 15020, NLM 15035, LPP 11111, LPP 11137, LPP 11145, LPP 11168, LPP 11195, LPP 11224, LPP 12051, LPP 12052, LPP 12062, LPP 12068, LPP 12103, LPP 12110, LPP 12137, NLH 12088, NLH 11211, NLH 11220, NLH 12159, NLH 12187, NLH 15003, NLH 17026, NLH 17039, NLI 17001, NLI 17002, NLI 17003, NLI 17057, NLI 17058, LHM 17006, LHM 17009, LHM 17019, LHM 17020, LHM 17021, LHM 17022, LHM 17023, LHM 17024, Masoor 85, Masoor 93, Shiraz- 96, NIA Masoor 05, Markaz 2009, Punjab Masoor 2019, GAEIL, ILL 6002, ILL 2245, ILL 2580, ILL 4400, ILL 8006, ILL 7978, X 2013-174-1, X 2011S-160-22, X 2011S 33-34-32, X 2011S-19-12, X 2011S-19-39 | 701-1000 | NLM 15014, NLM 15021, NLM 15025, NLM 15029, LPP 11025, NLH 12096, NLH 11229, NLH 12097, NLH 12196, NLH 15032, NLH 17034, NLI 17059, LHM 17008, LHM 17011, LHM 17013, LHM 17015, Mansehra- 89, Punjab Masoor 2020, NL 96621, NL 96635, NL 96700, Turk Masoor, Lentil Black, SEYRAN-96, ILL 18108, Precoz | 1001-2500 | NLM 15026, LPP 11001, LPP 12105, LPP 12182, NLI 15044, LHM 17010, LHM 17014, NIAB Masoor 2002, NIAB Masoor 2006, Punjab Masoor 2009, Chakwal Masoor, NL 96680, TCL 85-1, ALTINOPARK |  |  | |  | | |
| 6. | POD | ≤500 | | NLM 15011, NLM 15012, NLM 15014, NLM 15015, NLM 15016, NLM 15018, NLM 15019, NLM 15020, NLM 15021, NLM 15029, NLM 15035, LPP 11111, LPP 11137, LPP 11145, LPP 11168, LPP 11195, LPP 11224, LPP 12051, LPP 12052, LPP 12062, LPP 12068, LPP 12137, LPP 12182, NLH 12088, NLH 11211, NLH 11220, NLH 11229, NLH 12159, NLH 12187, NLH 12196, NLH 15003, NLH 15032, NLH 17026, NLH 17039, NLI 17003, NLI 17059, LHM 17006, LHM 17008, LHM 17009, LHM 17010, LHM 17011, LHM 17013, LHM 17014, LHM 17015, LHM 17019, LHM 17020, LHM 17021, LHM 17022, LHM 17023, LHM 17024, Masoor 85, Mansehra- 89, Masoor 93, Shiraz- 96, NIA Masoor 05, NIAB Masoor 2006, Punjab Masoor 2009, Chakwal Masoor, Punjab Masoor 2019, Punjab Masoor 2020, NL 96621, NL 96635, NL 96680, NL 96700, Turk Masoor, Lentil Black, TCL 85-1, ALTINOPARK, SEYRAN-96, GAEIL, Precoz, ILL 6002, ILL 2245, ILL 2580, ILL 4400, ILL 8006, ILL 7978, X 2013-174-1, X 2011S-160-22, X 2011S 33-34-32, X 2011S-19-12, X 2011S-19-39 | 501-1500 | LPP 11001, LPP 12103, LPP 12105, LPP 12110, NLH 12096, NLH 12097, NLH 17034, NLI 17001, NLI 17002, NLI 17058, Markaz 2009, ILL 18108 | 1501-3200 | NLM 15025, NLM 15026, LPP 11025, NLI 15044, NLI 17057, NIAB Masoor 2002 |  |  | |  | | |
| 7. | SOD | ≤140 | | NLM 15012, NLM 15016, LPP 12052, NLH 11229, NLH 12159, LHM 17006, LHM 17015, LHM 17019, Masoor 85, Mansehra- 89, Masoor 93, NIAB Masoor 2002, NIAB Masoor 2006, Markaz 2009, Punjab Masoor 2019, NL 96635, NL 96680, NL 96700, Turk Masoor, Lentil Black, TCL 85-1, ILL 6002, ILL 8006, ILL 7978, | 141-200 | NLM 15011, NLM 15018, NLM 15020, NLM 15025, NLM 15035, LPP 11025, LPP 11145, LPP 11168, LPP 12103, NLH 12096, NLH 12097, NLH 12196, NLH 15003, NLH 15032, NLH 17034, NLI 15044, NLI 17001, NLI 17002, NLI 17003, NLI 17057, LHM 17010, LHM 17011, LHM 17020, LHM 17022, LHM 17024, Shiraz- 96, Punjab Masoor 2009, Chakwal Masoor, Punjab Masoor 2020, NL 96621, SEYRAN-96, Precoz, X 2011S-160-22, | 201-296 | NLM 15014, NLM 15015, NLM 15019, NLM 15021, NLM 15026, NLM 15029, LPP 11001, LPP 11111, LPP 11137, LPP 11195, LPP 11224, LPP 12051, LPP 12062, LPP 12068, LPP 12105, LPP 12110, LPP 12137, LPP 12182, NLH 12088, NLH 11211, NLH 11220, NLH 12187, NLH 17026, NLH 17039, NLI 17058, NLI 17059, LHM 17008, LHM 17009, LHM 17013, LHM 17014, LHM 17021, LHM 17023, NIA Masoor 05, ALTINOPARK, GAEIL, ILL 18108, ILL 2245, ILL 2580, ILL 4400, X 2013-174-1, X 2011S 33-34-32, X 2011S-19-12, X 2011S-19-39, | | | | |  |  |
| 8. | PROT | ≤8000 | | NLM 15011, NLM 15012, NLM 15015, NLM 15016, NLM 15018, NLM 15019, NLM 15021, NLM 15035, LPP 11111, LPP 11137, LPP 11168, LPP 11195, LPP 12051, LPP 12052, NLH 12088, NLH 11211, NLH 15032, NLH 17026, LHM 17006, LHM 17014, LHM 17015, LHM 17021, Punjab Masoor 2020, ILL 6002, ILL 2245, ILL 7978, X 2011S-160-22 | 8001-10000 | NLM 15014, NLM 15020, NLM 15029, LPP 11145, LPP 11224, LPP 12062, LPP 12103, NLH 11220, NLH 11229, NLH 12159, NLH 12187, NLH 15003, NLH 17039, NLI 17057, LHM 17010, LHM 17011, LHM 17013, LHM 17019,LHM 17020, LHM 17022, LHM 17023, LHM 17024, Masoor 85, ALTINOPARK, SEYRAN-96, GAEIL, ILL 18108, ILL 2580, ILL 4400, ILL 8006, X 2013-174-1, X 2011S-19-12, X 2011S-19-39 | 10001-17000 | NLM 15025, NLM 15026, LPP 11001, LPP 11025, LPP 12068, LPP 12105, LPP 12110, LPP 12137, LPP 12182, NLH 12096, NLH 12097, NLH 12196, NLH 17034, NLI 15044, NLI 17001, NLI 17002, NLI 17003, NLI 17058, NLI 17059, LHM 17008, LHM 17009, Mansehra- 89, Masoor 93, Shiraz- 96, NIAB Masoor 2002, NIA Masoor 05, NIAB Masoor 2006, Markaz 2009, Punjab Masoor 2009, Chakwal Masoor, Punjab Masoor 2019, NL 96621, NL 96635, NL 96680, NL 96700, Turk Masoor, Lentil Black, TCL 85-1, Precoz, X 2011S 33-34-32, | | | | |  |  |
| 9. | Amylase | ≤100 | | NLM 15011, NLM 15012, NLM 15015, NLM 15016, NLM 15018, NLM 15021, NLM 15029, NLM 15035, LPP 11137, LPP 11145, LPP 11168, LPP 11195, LPP 11224, LPP 12051, LPP 12052, LPP 12062, NLH 12088, NLH 11211, NLH 11220, NLH 11229, NLH 12159, NLH 15032, NLH 17026, LHM 17006, LHM 17011, LHM 17019, LHM 17021, LHM 17022, LHM 17023, LHM 17024, Punjab Masoor 2020, SEYRAN-96, ALTINOPARK, GAEIL, ILL 6002, ILL 2580, ILL 4400, X 2013-174-1, X 2011S-160-22, X 2011S 33-34-32, X 2011S-19-12, X 2011S-19-39 | 101-150 | NLM 15014, NLM 15019, NLM 15025, NLM 15026, LPP 11001, LPP 11025, LPP 11111, LPP 12068, LPP 12103, LPP 12105, LPP 12110, LPP 12182, NLH 12096, NLH 12097, NLH 12187, NLH 12196, NLH 15003, NLH 17034, NLH 17039, NLI 15044, NLI 17001, NLI 17002, NLI 17003, NLI 17057, NLI 17058, NLI 17059, LHM 17008, LHM 17009, LHM 17010, LHM 17014, LHM 17015, Masoor 85, Mansehra- 89, Masoor 93, Shiraz- 96, NIAB Masoor 2002, NIA Masoor 05, NIAB Masoor 2006, Markaz 2009, Punjab Masoor 2009, Chakwal Masoor, Punjab Masoor 2019, NL 96621, NL 96635, NL 96680, NL 96700, Turk Masoor, Lentil Black, TCL 85-1, ILL 18108, Precoz, ILL 2245, ILL 8006, ILL 7978 | 151-273 | NLM 15020, LPP 12137, LHM 17013, LHM 17020 |  |  | |  | | |
| 10. | MDA | ≤160 | | NLM 15018, NLM 15019, NLM 15029, NLM 15035, LPP 11168, NLH 12187, NLH 17026, NLH 17034, LHM 17008, LHM 17011, LHM 17019, LHM 17020, LHM 17024, Chakwal Masoor, ILL 2245, X 2013-174-1, | 161-230 | NLM 15011, NLM 15012, NLM 15015, NLM 15016, NLM 15020, NLM 15021, NLM 15026, LPP 11111, LPP 11137, LPP 11145, LPP 11195, LPP 11224, LPP 12051, LPP 12052, LPP 12062, LPP 12068, LPP 12103, LPP 12137, LPP 12182, NLH 12096, NLH 11211, NLH 11220, NLH 11229, NLH 12097, NLH 12159, NLH 15032, NLI 17001, NLI 17002, NLI 17057, NLI 17058, LHM 17006, LHM 17009, LHM 17010, LHM 17013, LHM 17014, LHM 17015, LHM 17021, LHM 17022, LHM 17023, Masoor 85, Mansehra- 89, NIA Masoor 05, Markaz 2009, Punjab Masoor 2020, NL 96635, NL 96680, NL 96700, ALTINOPARK, SEYRAN-96, GAEIL, Precoz, ILL 6002, ILL 2580, ILL 4400, ILL 8006, ILL 7978, X 2011S-160-22, X 2011S 33-34-32, X 2011S-19-12, X 2011S-19-39 | 231-301 | NLM 15014, NLM 15025, LPP 11001, LPP 11025, LPP 12105, LPP 12110, NLH 12088, NLH 12196, NLH 15003, NLH 17039, NLI 15044, NLI 17003, NLI 17059, Masoor 93, Shiraz- 96, NIAB Masoor 2002, NIAB Masoor 2006, Punjab Masoor 2009, Punjab Masoor 2019, NL 96621, Turk Masoor, Lentil Black, TCL 85-1, ILL 18108, | | | | |  |  |
| 11. | TOS | ≤5000 | | NLM 15014, NLM 15025, NLM 15026, LPP 12068, LPP 12103, LPP 12105, LPP 12110, LPP 12137, LPP 12182, NLH 12096, NLH 12196, NLH 15003, NLH 17034, NLH 17039, NLI 15044, NLI 17001, NLI 17002, NLI 17003, NLI 17057, NLI 17058, NLI 17059, LHM 17008, LHM 17009, LHM 17010, Masoor 85, Mansehra- 89, Masoor 93, Shiraz- 96, NIAB Masoor 2002, NIA Masoor 05, NIAB Masoor 2006, Markaz 2009, Punjab Masoor 2009, Chakwal Masoor, Punjab Masoor 2019, NL 96621, NL 96635, NL 96680, NL 96700, Turk Masoor, Lentil Black, TCL 85-1, Precoz | 5001-15000 | NLM 15012, NLM 15015, NLM 15018, NLM 15020, NLM 15021, NLM 15035, LPP 11001, LPP 11025, LPP 11111, LPP 11137, LPP 11145, LPP 11168, LPP 11195, LPP 11224, LPP 12051, LPP 12052, LPP 12062, NLH 12088, NLH 11211, NLH 11220, NLH 11229, NLH 12097, NLH 12159, NLH 12187, NLH 15032, NLH 17026, LHM 17006, LHM 17013, LHM 17014, LHM 17019, LHM 17020, LHM 17021, LHM 17023, LHM 17024, Punjab Masoor 2020, GAEIL,ILL 18108, ILL 6002, ILL 2580, ILL 8006 | 15001-48000 | NLM 15011, NLM 15016, NLM 15019, NLM 15029, LHM 17011, LHM 17015, LHM 17022, ALTINOPARK, SEYRAN-96, ILL 2245, ILL 4400, ILL 7978, X 2013-174-1, X 2011S-160-22, X 2011S 33-34-32, X 2011S-19-12, X 2011S-19-39 |  |  | |  | | |
| 12. | TSP | ≤200 | | LPP 11001, LPP 11025, LPP 12068, LPP 12103, LPP 12105, LPP 12110, LPP 12137, LPP 12182, NLH 17039, NLI 17001, NLI 17059, LHM 17008, LHM 17009, LHM 17010, NIA Masoor 05, Punjab Masoor 2009, Chakwal Masoor, Punjab Masoor 2020, NL 96621, NL 96700, TCL 85-1, Lentil Black, ILL 18108, Precoz | 201-350 | NLM 15014, NLM 15025, NLM 15035, NLH 12097, NLH 12196, NLH 15032, NLH 17034, NLI 17002, NLI 17003, NLI 17057, Mansehra- 89, Masoor 93, NIAB Masoor 2002, Markaz 2009, Punjab Masoor 2019, NL 96635, Turk Masoor, X 2011S-160-22 | 351-548 | NLM 15011, NLM 15012, NLM 15015, NLM 15016, NLM 15018, NLM 15019, NLM 15020, NLM 15021, NLM 15026, NLM 15029, LPP 11111, LPP 11137, LPP 11145, LPP 11168, LPP 11195, LPP 11224, LPP 12051, LPP 12052, LPP 12062, NLH 12088, NLH 12096, NLH 11211, NLH 11220, NLH 11229, NLH 12159, NLH 12187, NLH 15003, NLH 17026, NLI 15044, NLI 17058, LHM 17006, LHM 17011, LHM 17013, LHM 17014, LHM 17015, LHM 17019, LHM 17020, LHM 17021, LHM 17022, LHM 17023, LHM 17024, Masoor 85, Shiraz- 96, NIAB Masoor 2006, NL 96680, ALTINOPARK, SEYRAN-96, GAEIL, ILL 6002, ILL 2245, ILL 2580, ILL 4400, ILL 8006, ILL 7978, X 2013-174-1, X 2011S 33-34-32, X 2011S-19-12, X 2011S-19-39 | | | | |  |  |
| 13. | TSS | ≤30 | | NLM 15011, NLM 15018, LPP 11111, LPP 12051, LPP 12052, NLH 11220, LHM 17011, LHM 17014, LHM 17023, Chakwal Masoor, Punjab Masoor 2019, Lentil Black, GAEIL, X 2011S-160-22, X 2011S 33-34-32, X 2011S-19-39 | 31-50 | NLM 15012, NLM 15014, NLM 15019, NLM 15021, NLM 15025, NLM 15026, NLM 15029, NLM 15035, LPP 11001, LPP 11025, LPP 11145, LPP 11168, LPP 11224, LPP 12068, LPP 12103, LPP 12105, LPP 12110, LPP 12137, LPP 12182, NLH 12088, NLH 12096, NLH 11229, NLH 12097, NLH 12196, NLH 15003, NLH 15032, NLH 17026, NLH 17034, NLI 15044, NLI 17002, NLI 17003, NLI 17057, NLI 17058, NLI 17059, LHM 17006, LHM 17008, LHM 17009, LHM 17010, LHM 17013, LHM 17015, LHM 17021, LHM 17022, LHM 17024, Masoor 85, Mansehra- 89, Masoor 93, Shiraz- 96, NIAB Masoor 2002, NIA Masoor 05, NIAB Masoor 2006, Punjab Masoor 2020, NL 96621, NL 96635, NL 96680, NL 96700, Turk Masoor, TCL 85-1, SEYRAN-96, ILL 18108, ILL 2245, ILL 2580, ILL 4400, X 2013-174-1, X 2011S-19-12 | 51-85 | NLM 15015, NLM 15016, NLM 15020, LPP 11137, LPP 11195, LPP 12062, NLH 11211, NLH 12159, NLH 12187, NLH 17039, NLI 17001, LHM 17019, LHM 17020, Markaz 2009, Punjab Masoor 2009, ALTINOPARK, Precoz, ILL 6002, ILL 8006, ILL 7978, | | | | |  |  |
| 14. | TAC | ≤5 | | NLM 15014, NLM 15015, NLM 15020, NLM 15021, NLM 15025, LPP 11025, LPP 11137, LPP 11145, LPP 11195, LPP 11224, LPP 12051, LPP 12052, LPP 12062, LPP 12068, LPP 12110, LPP 12182, NLH 12088, NLH 11211, NLH 11220, NLH 11229, NLH 12187, NLH 12196, NLH 15003, NLH 15032, NLH 17026, NLH 17039, NLI 17001, NLI 17002, NLI 17057, NLI 17058, NLI 17059, LHM 17006, LHM 17008, LHM 17010, LHM 17011, LHM 17013, LHM 17019, LHM 17020, LHM 17021, LHM 17023, Mansehra- 89, Masoor 93, Shiraz- 96, NIAB Masoor 2006, Markaz 2009, NL 96621, NL 96700, Lentil Black, TCL 85-1, SEYRAN-96, ILL 18108, ILL 2580, ILL 8006, ILL 7978, X 2011S 33-34-32, | 5.01-10 | NLM 15011, NLM 15012, NLM 15018, NLM 15019, NLM 15026, NLM 15029, NLM 15035, LPP 11001, LPP 11111, LPP 11168, LPP 12103, LPP 12105, LPP 12137, NLH 12096, NLH 12097, NLH 12159, NLH 17034, NLI 15044, NLI 17003, LHM 17014, LHM 17015, LHM 17022, LHM 17024, NIAB Masoor 2002, Punjab Masoor 2009, Chakwal Masoor, Punjab Masoor 2019, Punjab Masoor 2020, NL 96635, Turk Masoor, GAEIL, ILL 6002, ILL 2245, ILL 4400, X 2011S-160-22, X 2011S-19-12, | 10.1-16 | NLM 15016, LHM 17009, Masoor 85, NIA Masoor 05, NL 96680, ALTINOPARK, Precoz, X 2013-174-1 |  |  | |  | | |
| 15. | NRS | ≤20 | | NLM 15011, NLM 15018, NLM 15021, LPP 11111, LPP 12051, LPP 12052, NLH 11220, NLH 17026, NLI 17057, LHM 17011, LHM 17014, LHM 17023, Chakwal Masoor, Punjab Masoor 2019, Turk Masoor, Lentil Black, GAEIL, ILL 8006, X 2011S-160-22, X 2011S 33-34-32, X 2011S-19-39 | 20.1-50 | NLM 15012, NLM 15014, NLM 15016, NLM 15019, NLM 15020, NLM 15021, NLM 15025, NLM 15026, NLM 15029, NLM 15035, LPP 11001, LPP 11025, LPP 11137, LPP 11145, LPP 11168, LPP 11195, LPP 11224, LPP 12062, LPP 12068, LPP 12103, LPP 12105, LPP 12110, LPP 12137, LPP 12182, NLH 12088, NLH 12096, NLH 11229, NLH 12097, NLH 12159, NLH 12187, NLH 12196, NLH 15003, NLH 15032, NLH 17034, NLI 15044, NLI 17001, NLI 17002, NLI 17003, NLI 17058, NLI 17059, LHM 17006, LHM 17008, LHM 17009, LHM 17010, LHM 17013, LHM 17015, LHM 17019, LHM 17020, LHM 17021, LHM 17022, LHM 17024, Masoor 85, Mansehra- 89, Masoor 93, Shiraz- 96, NIAB Masoor 2002, NIA Masoor 05, NIAB Masoor 2006, Punjab Masoor 2020, NL 96621, NL 96635, NL 96680, NL 96700, TCL 85-1, SEYRAN-96, ILL 18108, ILL 6002, ILL 2245, ILL 2580, ILL 4400, X 2013-174-1, X 2011S-19-12, | 50.1-75 | NLM 15015, NLH 11211, NLH 17039, Punjab Masoor 2009, ALTINOPARK, Precoz, ILL 7978 |  |  | |  | | |
| 16. | RS | ≤10 | | NLM 15014, NLM 15015, NLM 15016, NLM 15018, NLM 15019, NLM 15025, NLM 15026, NLM 15035, LPP 11025, LPP 11111, LPP 11137, LPP 11168, LPP 11195, LPP 11224, LPP 12051, LPP 12052, LPP 12062, LPP 12068, LPP 12103, LPP 12105, LPP 12110, LPP 12137, LPP 12182, NLH 12088, NLH 12096, NLH 11211, NLH 11220, NLH 11229, NLH 12097, NLH 12196, NLH 15003, NLH 15032, NLH 17034, NLH 17039, NLI 15044, NLI 17001, NLI 17002, NLI 17003, NLI 17058, NLI 17059, LHM 17008, LHM 17009, LHM 17010, LHM 17013, LHM 17014, LHM 17015, LHM 17021, LHM 17022, LHM 17023, Masoor 85, Mansehra- 89, Masoor 93, Shiraz- 96, NIAB Masoor 2002, NIA Masoor 05, NIAB Masoor 2006, Markaz 2009, Punjab Masoor 2009, Chakwal Masoor, Punjab Masoor 2019, NL 96621, NL 96635, NL 96680, NL 96700, Lentil Black, TCL 85-1, ALTINOPARK, SEYRAN-96, GAEIL, ILL 18108, Precoz, ILL 6002, ILL 2245, ILL 4400, ILL 7978, X 2011S-19-39 | 10.1-30 | NLM 15011, NLM 15012, NLM 15020, NLM 15021, NLM 15029, LPP 11001, LPP 11145, NLH 12159, NLH 17026, NLI 17057, LHM 17006, LHM 17011, LHM 17019, LHM 17020, LHM 17024, Punjab Masoor 2020, ILL 2580, X 2013-174-1, X 2011S-160-22, X 2011S 33-34-32, X 2011S-19-12, | 30.1-46 | NLH 12187, Turk Masoor, ILL 8006 |  |  | |  | | |
| 17. | Lycopene | ≤5 | | NLM 15011, NLM 15012, NLM 15014, NLM 15015, NLM 15016, NLM 15018, NLM 15019, NLM 15020, NLM 15021, NLM 15026, NLM 15029, NLM 15035, LPP 11001, LPP 11111, LPP 11137, LPP 11145, LPP 11168, LPP 11195, LPP 11224, LPP 12051, LPP 12052, LPP 12062, LPP 12068, LPP 12105, LPP 12110, LPP 12137, LPP 12182, NLH 12088, NLH 12096, NLH 11211, NLH 11220, NLH 11229, NLH 12159, NLH 12187, NLH 15032, NLH 17026, NLI 17058, NLI 17059, LHM 17006, LHM 17008, LHM 17009, LHM 17011, LHM 17013, LHM 17014, LHM 17015, LHM 17019, LHM 17020, LHM 17021, LHM 17022, LHM 17023, LHM 17024, NIA Masoor 05, Punjab Masoor 2020, ALTINOPARK, SEYRAN 96, GAEIL, ILL 6002, ILL 2245, ILL 2580, ILL 4400, ILL 8006, ILL 7978, X 2013-174-1, X 2011S-160-22, X 2011S 33-34-32, X 2011S-19-12, X 2011S-19-39 | 5.0-9.99 | NLM 15025, LPP 11025, LPP 12103, NLH 12196, NLH 15003, NLH 17034, NLH 17039, NLI 15044, NLI 17001, NLI 17002, NLI 17003, NLI 17057, LHM 17010, Masoor 85, Mansehra 89, Masoor 93, Shiraz 96, NIAB Masoor 2002, NIAB Masoor 2006, Punjab Masoor 2009, Chakwaal Masoor, Punjab Masoor 2019, NL 96621, NL 96635, NL 96680, NL 96700, Lentil Black, TCL 85-1, ILL 18108, Precoz | 10.00-11.00 | NLH 12097, Markaz 2009, Turk Masoor |  |  | |  | | |
| 18. | Chl. A | ≤100 | | NLM 15011, NLM 15015, NLM 15016, NLM 15018, NLM 15019, NLM 15020, NLM 15021, NLM 15029, NLM 15035, LPP 11001, LPP 11111, LPP 11137, LPP 11145, LPP 11168, LPP 11195, LPP 11224, LPP 12051, LPP 12052, LPP 12062, LPP 12105, LPP 12137, LPP 12182, NLH 12088, NLH 12096, NLH 11211, NLH 11220, NLH 11229, NLH 12159, NLH 15032, NLH 17026, NLI 17057, LHM 17006, LHM 17013, LHM 17014, LHM 17015, LHM 17019, LHM 17020, LHM 17021, LHM 17022, LHM 17023, LHM 17024, NIA Masoor 05, Punjab Masoor 2020, ALTINOPARK, SEYRAN- 96, GAEIL, ILL 6002, ILL 2245, ILL 2580, ILL 4400, ILL 8006, ILL 7978, X 2013-174-1, X 2011S-160-22, X 2011S 33-34-32, X 2011S-19-12, X 2011S-19-39 | 100.01-200 | NLM 15012, NLM 15014, NLM 15025, NLM 15026, LPP 11025, LPP 12068, LPP 12103, LPP 12110, NLH 12097, NLH 12187, NLH 12196, NLH 15003, NLH 15034, NLH 15039, NLI 15044, NLI 17001, NLI 17002, NLI 17003, NLI 17058, NLI 17059, LHM 17008, LHM 17009, LHM 17010, Masoor 85, Mansehra 89, Masoor 93, Shiraz 96, NIAB Masoor 2006, Markaz 2009, Punjab Masoor 2009, Chakwal Masoor, Punjab Masoor 2019, NL 96621, NL 96635, NL 96680, NL 96700, Lentil Black, TCL 85-1, ILL 18108, Precoz | 200.01-240 | NLH 12097, NIAB Masoor 2002, Turk Masoor |  |  | |  | | |
| 19. | Chl. B | ≤100 | | NLM 15011, NLM 15014, NLM 15015, NLM 15019, NLM 15020, NLM 15026, NLM 15035, LPP 11001, LPP 11111, LPP 11137, LPP 11145, LPP 11168, LPP 11195, LPP 12051, LPP 12062, LPP 12068, LPP 12105, LPP 12137, LPP 12182, NLH 12088, NLH 12096, NLH 11211, NLH 11229, NLH 12159, NLH 15032, NLH 17026, LHM 17006, LHM 17008, LHM 17009, LHM 17014, LHM 17015, LHM 17019, LHM 17020, LHM 17021, LHM 17022, LHM 17023, LHM 17024, NIA Masoor 05, Punjab Masoor 2020, ALTINOPARK, SEYRAN-96, GAEIL, ILL 6002, ILL 2245, ILL 2580, ILL 4400, ILL 8006, ILL 7978, X 2013-174-1, X 2011S-160-22, X 2011S 33-34-32, X 2011S-19-12, X 2011S-19-39 | 100.01-200 | NLM 15012, NLM 15016, NLM 15018, NLM 15021, NLM 15025, NLM 15029, LPP 11224, LPP 12052, LPP 12103, LPP 12110, NLH 11220, NLH 12187, NLH 12196, NLH 15003, NLH 17034, NLH 17039, NLI 15044, NLI 17001, NLI 17002, NLI 17003, NLI 17057, NLI 17058, NLI 17059, LHM 17010, LHM 17011, LHM 17013, Shiraz-96, NL 96621, NL 96635, NL 96700, TCL 85-1 | 200.01- 320 | LPP 11025, NLH 12097, Masoor 85, Mansehra 89, Masoor 93, NIAB Masoor 2002, NIAB Masoor 2006, Markaz 2009, Punjab Masoor 2009, Chakwal Masoor, Punjab Masoor 2019, NL 96680, Turk Masoor, Lentil Black, ILL 18108, Precoz | | |  | | |  |
| 20. | Total chl. | ≤150 | | NLM 15011, NLM 15015, NLM 15018, NLM 15019, LPP 11111, LPP 11137, LPP 11145, LPP 11168, LPP 11195, LPP 12051, LPP 12062, NLH 12088, NLH 12096, NLH 11211, NLH 12159, NLH 15032, NLH 17026, LHM 17006, LHM 17014, LHM 17019, LHM 17020, LHM 17021, LHM 17022, LHM 17023, LHM 17024, NIA Masoor 05, NIAB Masoor 2020, ALTINOPARK, SEYRAN-96, GAEIL, ILL 6002, ILL 2245, ILL 2580, ILL 4400, ILL 8006, ILL 7978, X 2013-174-1, X 2011S-160-22, X 2011S 33-34-32, X 2011S-19-12, X 2011S-19-39 | 150.01-250 | NLM 15012, NLM 15014, NLM 15016, NLM 15020, NLM 15021, NLM 15025, NLM 15026, NLM 15029, NLM 15035, LPP 11001, LPP 11224, LPP 12052, LPP 12068, LPP 12182, NLH 12103, NLH 12105, NLH 12110, NLH 12137, NLI 15044, NLI 17057, NLI 17058, LHM 17008, LHM 17009, LHM 17010, LHM 17013, LHM 17015, NIA Masoor 05 | 250.01-553 | LPP 11025, NLH 12097, NLH 12159, NLH 12187, NLH 12196, NLH 15003, NLH 17034, NLH 17039, NLI 17001, NLI 17002, NLI 17003, LHM 17011, Masoor 85, Mansehra 89, Masoor 93, Shiraz-96, NIAB Masoor 2002, NIA Masoor 05, NIAB Masoor 2006, Markaz 2009, NIAB Masoor 2009, Chakwal Masoor, Punjab Masoor 2019, NL 96621, NL 96635, NL 96700, NL 96680, Turk Masoor, Lentil Black, TCL 85-1, ILL 18108, Precoz | | | | |  |  |
| 21. | Total carotenoids | ≤ 5 | | NLM 15011, NLM 15015, NLM 15016, NLM 15018, NLM 15019, NLM 15020, NLM 15035, LPP 11111, LPP 11137, LPP 11145, LPP 11168, LPP 11195, LPP 12051, LPP 12062, NLH 12088, NLH 12096, NLH 11211, NLH 11229, NLH 12159, NLH 15032, NLH 17026, LHM 17006, LHM 17013, LHM 17014, LHM 17019, LHM 17020, LHM 17021, LHM 17022, LHM 17023, LHM 17024, Punjab Masoor 2020, ALTINOPARK, SEYRAN-96, GAEIL, ILL 6002, ILL 2245, ILL 2580, ILL 4400, ILL 8006, ILL 7978, X 2011S-160-22, X 2011S 33-34-32, X 2011S-19-12, X 2011S-19-39 | 5.01-10.0 | NLM 15012, NLM 15021, NLM 15026, NLM 15029, LPP 11224, LPP 12052, LPP 12103, LPP 12105, LPP 12110, LPP 12137, LPP 12182, NLH 11220, NLH 12187, NLI 17057, NLI 17058, NLI 17059, LHM 17008, LHM 17009, LHM 17011, LHM 17015, NIA Masoor 05, X 2013-174-1 | 10.01-18 | NLM 15014, NLM 15025, LPP 11001, LPP 11025, LPP 12068, NLH 12097, NLH 12196, NLH 15003, NLH 17034, NLH 17039, NLI 15044, NLI 17001, NLI 17002, NLI 17003, LHM 17010, Masoor 85, Mansehra-89, Masoor 93, Shiraz-96, NIAB Masoor 2002, NIAB Masoor 2006, Markaz 2009, Punjab Masoor 2009, Punjab Masoor 2019, Chakwal Masoor, NL 96621, NL 96635, NL 96680, NL 96700, Turk Masoor, Lentil Black, TCL 85-1, ILL 18108, Precoz | | | | |  |  |
| 22. | TFs | ≤ 150 | | NLM 15011, NLM 15012, NLM 15015, NLM 15016, NLM 15018, NLM 15019, NLM 15020, NLM 15021, NLM 15029, NLM 15035, LPP 11137, LPP 11111, LPP 11224, LPP 11168, LPP 11145, LPP 11195, LPP 12051, LPP 12052, LPP 12062,NLH 12088, NLH 11211, NLH 11220, NLH 11229, NLH 12159, NLH 12187, NLH 15032, NLH 17026, LHM 17006, LHM 17011, LHM 17013, LHM 17014, LHM 17015, LHM 17019, LHM 17020, LHM 17021, LHM 17022, LHM 17023, LHM 17024, Punjab Masoor 2020, ALTINOPARK, SEYRAN-96, GAEIL, ILL 6002, ILL 2245, ILL 2580, ILL 4400, ILL 8006, ILL 7978, X 2013-174-1, X 2011S-160-22, X 2011S 33-34-32, X 2011S-19-12, X 2011S-19-39 | 150.01-300 | NLM 15025, LPP 12137, NLH 17034, NLH 17039, NLI 17002, NLI 17003, NLI 17058, Masoor 93, NIAB Masoor 2002, NIAB Masoor 2006, Markaz 2009, Chakwal Masoor, NL 96700, Lentil Black | 300.01-367 | NLM 15014, NLM 15026, LPP 11001, LPP 11025, LPP 12068, LPP 12103, LPP 12105, LPP 12110, LPP 12182, NLH 12096, NLH 12097, NLH 12196, NLH 15003, NLI 15044, NLI 17001, NLI 17057, NLI 17059, LHM 17008, LHM 17009, LHM 17010, Masoor 85, Mansehra-89, Shiraz-96, NIA Masoor 05, Punjab Masoor 2009, Punjab Masoor 2019, NL 96621, NL 96635, NL 96680, Turk Masoor, TCL 85-1, ILL 18108, Precoz | | | | |  |  |

**Table S2.** Descriptive statistics of the groups of lentil genotypes for studied biochemical traits.

| **Statistic** | **Minimum** | **Maximum** | **Range** | **Mean** | **Variance (n-1)** | **Standard deviation (n-1)** | **Standard error of the mean** | **Standard error of the variance** |
| --- | --- | --- | --- | --- | --- | --- | --- | --- |
| **TPC(Mean) \| G-M** | 8840.000 | 43680.000 | 34840.000 | 18540.071 | 105393115.879 | 10266.115 | 2743.735 | 41338581.113 |
| **TOS(Mean) \| G-M** | 970.000 | 47487.500 | 46517.500 | 12971.786 | 159073085.989 | 12612.418 | 3370.810 | 62393597.657 |
| **Protease(Mean \| G-M** | 6300.000 | 14135.000 | 7835.000 | 8696.964 | 5584327.710 | 2363.118 | 631.570 | 2190353.536 |
| **Tanins(Mean) \| G-M** | 1745.000 | 13950.000 | 12205.000 | 7805.000 | 12963366.346 | 3600.468 | 962.265 | 5084650.612 |
| **AsA(Mean) \| G-M** | 610.750 | 682.500 | 71.750 | 634.918 | 446.571 | 21.132 | 5.648 | 175.160 |
| **(APX)Mean \| G-M** | 41.500 | 1195.000 | 1153.500 | 514.750 | 160914.837 | 401.142 | 107.210 | 63115.992 |
| **CAT(Mean) \| G-M** | 30.000 | 3740.000 | 3710.000 | 417.321 | 922942.754 | 960.699 | 256.758 | 362007.932 |
| **POD(Mean) \| G-M** | 63.700 | 2174.700 | 2111.000 | 385.200 | 432063.715 | 657.316 | 175.675 | 169469.332 |
| **TSP(Mean) \| G-M** | 152.500 | 464.333 | 311.833 | 378.557 | 8981.360 | 94.770 | 25.328 | 3522.779 |
| **SOD(Mean) \| G-M** | 103.739 | 278.671 | 174.932 | 188.300 | 2786.738 | 52.790 | 14.109 | 1093.049 |
| **MDA(Mean) \| G-M** | 138.226 | 275.161 | 136.935 | 187.424 | 1691.556 | 41.129 | 10.992 | 663.483 |
| **TOTAL CHLORO(Mean) \| G-M** | 39.236 | 288.454 | 249.218 | 172.575 | 5213.517 | 72.205 | 19.298 | 2044.909 |
| **(TF)Mean \| G-M** | 66.416 | 362.797 | 296.381 | 153.051 | 13122.715 | 114.554 | 30.616 | 5147.152 |
| **Amylase(Mean) \| G-M** | 60.547 | 233.698 | 173.151 | 98.682 | 1941.927 | 44.067 | 11.777 | 761.686 |
| **CHLB(Mean) \| G-M** | 13.245 | 197.678 | 184.433 | 96.437 | 2338.108 | 48.354 | 12.923 | 917.082 |
| **CHLA(Mean) \| G-M** | 29.691 | 115.530 | 85.840 | 81.273 | 862.812 | 29.374 | 7.850 | 338.423 |
| **TS(Mean) \| G-M** | 13.720 | 83.936 | 70.216 | 44.803 | 256.285 | 16.009 | 4.279 | 100.523 |
| **NRS(Mean) \| G-M** | 4.843 | 74.797 | 69.954 | 36.217 | 254.346 | 15.948 | 4.262 | 99.763 |
| **RS(Mean) \| G-M** | 4.690 | 20.629 | 15.939 | 9.069 | 17.247 | 4.153 | 1.110 | 6.765 |
| **TOTAL CAR(Mean) \| G-M** | 2.455 | 13.568 | 11.113 | 6.506 | 12.433 | 3.526 | 0.942 | 4.877 |
| **TAC(Mean) \| G-M** | 0.377 | 15.763 | 15.386 | 5.842 | 17.757 | 4.214 | 1.126 | 6.965 |
| **LYCO(Mean) \| G-M** | 0.889 | 7.489 | 6.600 | 3.495 | 2.954 | 1.719 | 0.459 | 1.159 |
| **TPC(Mean) \| G-R** | 6457.500 | 45287.500 | 38830.000 | 19603.538 | 124728711.758 | 11168.201 | 2190.264 | 35278607.157 |
| **Tanins(Mean) \| G-R** | 257.500 | 13512.500 | 13255.000 | 9113.077 | 14771370.214 | 3843.354 | 753.744 | 4177974.418 |
| **Protease(Mean \| G-R** | 6125.000 | 13985.000 | 7860.000 | 9092.154 | 5063708.015 | 2250.268 | 441.314 | 1432232.910 |
| **TOS(Mean) \| G-R** | 193.500 | 13807.500 | 13614.000 | 7264.654 | 18479463.275 | 4298.775 | 843.059 | 5226781.518 |
| **CAT(Mean) \| G-R** | 90.000 | 3040.000 | 2950.000 | 857.835 | 722180.191 | 849.812 | 166.662 | 204263.404 |
| **AsA(Mean) \| G-R** | 596.500 | 706.500 | 110.000 | 632.827 | 575.409 | 23.988 | 4.704 | 162.750 |
| **(APX)Mean \| G-R** | 83.000 | 1710.000 | 1627.000 | 603.962 | 120695.738 | 347.413 | 68.133 | 34137.910 |
| **TSP(Mean) \| G-R** | 75.627 | 458.783 | 383.156 | 346.785 | 15809.170 | 125.735 | 24.659 | 4471.508 |
| **POD(Mean) \| G-R** | 63.300 | 1690.800 | 1627.500 | 252.975 | 146427.037 | 382.658 | 75.045 | 41415.820 |
| **SOD(Mean) \| G-R** | 118.313 | 294.046 | 175.733 | 214.361 | 3004.432 | 54.813 | 10.750 | 849.782 |
| **MDA(Mean) \| G-R** | 97.823 | 259.645 | 161.823 | 195.980 | 1397.990 | 37.390 | 7.333 | 395.411 |
| **(TF)Mean \| G-R** | 57.362 | 341.696 | 284.334 | 177.266 | 11387.958 | 106.714 | 20.928 | 3221.001 |
| **TOTAL CHLORO(Mean) \| G-R** | 35.192 | 429.082 | 393.890 | 160.612 | 11867.032 | 108.936 | 21.364 | 3356.504 |
| **Amylase(Mean) \| G-R** | 55.604 | 271.877 | 216.274 | 98.176 | 1774.849 | 42.129 | 8.262 | 502.003 |
| **CHLB(Mean) \| G-R** | 8.395 | 286.316 | 277.921 | 84.266 | 4765.143 | 69.030 | 13.538 | 1347.786 |
| **CHLA(Mean) \| G-R** | 26.493 | 173.671 | 147.178 | 75.543 | 1756.551 | 41.911 | 8.219 | 496.828 |
| **TS(Mean) \| G-R** | 13.491 | 70.909 | 57.417 | 44.247 | 173.129 | 13.158 | 2.580 | 48.968 |
| **NRS(Mean) \| G-R** | 6.124 | 55.864 | 49.740 | 33.910 | 185.506 | 13.620 | 2.671 | 52.469 |
| **RS(Mean) \| G-R** | 2.928 | 36.354 | 33.426 | 9.755 | 64.754 | 8.047 | 1.578 | 18.315 |
| **TOTAL CAR(Mean) \| G-R** | 1.696 | 17.119 | 15.423 | 6.633 | 20.376 | 4.514 | 0.885 | 5.763 |
| **LYCO(Mean) \| G-R** | 0.608 | 9.788 | 9.180 | 3.452 | 5.906 | 2.430 | 0.477 | 1.670 |
| **TAC(Mean) \| G-R** | 0.331 | 7.732 | 7.401 | 2.707 | 6.351 | 2.520 | 0.494 | 1.796 |
| **TPC(Mean) \| G-BCR** | 14275.000 | 44365.000 | 30090.000 | 31496.000 | 138228420.625 | 11757.058 | 5257.916 | 97742253.577 |
| **Protease(Mean \| G-BCR** | 8372.500 | 13156.000 | 4783.500 | 10892.700 | 3353162.700 | 1831.164 | 818.922 | 2371044.084 |
| **Tanins(Mean) \| G-BCR** | 3205.000 | 16125.000 | 12920.000 | 8797.500 | 28454243.750 | 5334.252 | 2385.550 | 20120188.709 |
| **TOS(Mean) \| G-BCR** | 96.000 | 12660.000 | 12564.000 | 3395.200 | 29188287.700 | 5402.619 | 2416.124 | 20639236.164 |
| **CAT(Mean) \| G-BCR** | 132.500 | 5595.000 | 5462.500 | 1925.000 | 4921434.375 | 2218.431 | 992.112 | 3479979.620 |
| **POD(Mean) \| G-BCR** | 617.300 | 1168.100 | 550.800 | 827.390 | 43468.122 | 208.490 | 93.240 | 30736.604 |
| **(APX)Mean \| G-BCR** | 495.000 | 1085.000 | 590.000 | 749.500 | 48657.500 | 220.584 | 98.648 | 34406.048 |
| **AsA(Mean) \| G-BCR** | 564.000 | 700.250 | 136.250 | 638.450 | 3187.138 | 56.455 | 25.247 | 2253.647 |
| **(TF)Mean \| G-BCR** | 317.800 | 365.592 | 47.792 | 337.366 | 316.080 | 17.779 | 7.951 | 223.502 |
| **TOTAL CHLORO(Mean) \| G-BCR** | 37.853 | 552.586 | 514.734 | 241.918 | 35602.854 | 188.687 | 84.383 | 25175.019 |
| **MDA(Mean) \| G-BCR** | 163.968 | 292.177 | 128.210 | 222.923 | 2138.161 | 46.240 | 20.679 | 1511.908 |
| **SOD(Mean) \| G-BCR** | 170.332 | 296.750 | 126.418 | 219.834 | 2726.078 | 52.212 | 23.350 | 1927.628 |
| **TSP(Mean) \| G-BCR** | 80.000 | 383.771 | 303.771 | 184.787 | 14930.861 | 122.192 | 54.646 | 10557.713 |
| **CHLB(Mean) \| G-BCR** | 9.208 | 317.312 | 308.104 | 126.366 | 13095.700 | 114.436 | 51.178 | 9260.059 |
| **CHLA(Mean) \| G-BCR** | 28.320 | 236.124 | 207.804 | 116.100 | 5696.766 | 75.477 | 33.754 | 4028.222 |
| **Amylase(Mean) \| G-BCR** | 106.330 | 119.726 | 13.396 | 114.526 | 29.761 | 5.455 | 2.440 | 21.045 |
| **TS(Mean) \| G-BCR** | 37.815 | 49.519 | 11.704 | 43.444 | 26.624 | 5.160 | 2.308 | 18.826 |
| **NRS(Mean) \| G-BCR** | 32.273 | 44.628 | 12.355 | 38.003 | 25.501 | 5.050 | 2.258 | 18.032 |
| **TOTAL CAR(Mean) \| G-BCR** | 1.633 | 16.869 | 15.236 | 9.235 | 29.296 | 5.413 | 2.421 | 20.715 |
| **RS(Mean) \| G-BCR** | 4.532 | 6.949 | 2.417 | 5.551 | 0.892 | 0.945 | 0.422 | 0.631 |
| **LYCO(Mean) \| G-BCR** | 0.637 | 10.881 | 10.244 | 5.132 | 13.560 | 3.682 | 1.647 | 9.589 |
| **TAC(Mean) \| G-BCR** | 0.431 | 7.219 | 6.788 | 4.882 | 6.742 | 2.597 | 1.161 | 4.768 |
| **TPC(Mean) \| G-EG-I** | 9662.500 | 54562.500 | 44900.000 | 23938.800 | 215821897.458 | 14690.878 | 2938.176 | 62302415.297 |
| **Tanins(Mean) \| G-EG-I** | 4125.000 | 24563.000 | 20438.000 | 10295.020 | 21803491.364 | 4669.421 | 933.884 | 6294125.804 |
| **TOS(Mean) \| G-EG-I** | 48.000 | 24405.000 | 24357.000 | 9974.200 | 74709492.792 | 8643.465 | 1728.693 | 21566772.887 |
| **Protease(Mean \| G-EG-I** | 6445.000 | 14160.000 | 7715.000 | 9904.320 | 4549702.706 | 2133.003 | 426.601 | 1313386.041 |
| **CAT(Mean) \| G-EG-I** | 62.500 | 4150.000 | 4087.500 | 800.280 | 1164650.731 | 1079.190 | 215.838 | 336205.706 |
| **(APX)Mean \| G-EG-I** | 41.500 | 2465.500 | 2424.000 | 694.480 | 209155.760 | 457.336 | 91.467 | 60378.067 |
| **AsA(Mean) \| G-EG-I** | 606.250 | 689.500 | 83.250 | 636.850 | 498.307 | 22.323 | 4.465 | 143.849 |
| **POD(Mean) \| G-EG-I** | 63.600 | 2147.200 | 2083.600 | 371.255 | 351270.393 | 592.681 | 118.536 | 101403.028 |
| **TSP(Mean) \| G-EG-I** | 38.167 | 546.333 | 508.167 | 338.797 | 20465.891 | 143.059 | 28.612 | 5907.994 |
| **(TF)Mean \| G-EG-I** | 101.915 | 366.420 | 264.505 | 209.931 | 10313.528 | 101.556 | 20.311 | 2977.259 |
| **MDA(Mean) \| G-EG-I** | 155.097 | 260.968 | 105.871 | 200.223 | 1067.819 | 32.678 | 6.536 | 308.253 |
| **SOD(Mean) \| G-EG-I** | 106.124 | 285.218 | 179.095 | 193.614 | 2361.927 | 48.600 | 9.720 | 681.830 |
| **TOTAL CHLORO(Mean) \| G-EG-I** | 37.659 | 528.383 | 490.724 | 168.151 | 22738.279 | 150.792 | 30.158 | 6563.976 |
| **Amylase(Mean) \| G-EG-I** | 67.283 | 126.057 | 58.774 | 100.651 | 285.486 | 16.896 | 3.379 | 82.413 |
| **CHLB(Mean) \| G-EG-I** | 11.589 | 312.099 | 300.509 | 88.425 | 8313.746 | 91.180 | 18.236 | 2399.972 |
| **CHLA(Mean) \| G-EG-I** | 27.065 | 229.670 | 202.605 | 80.858 | 3716.567 | 60.964 | 12.193 | 1072.880 |
| **TS(Mean) \| G-EG-I** | 11.497 | 71.780 | 60.283 | 40.939 | 211.764 | 14.552 | 2.910 | 61.131 |
| **NRS(Mean) \| G-EG-I** | 3.695 | 67.195 | 63.499 | 29.094 | 288.148 | 16.975 | 3.395 | 83.181 |
| **RS(Mean) \| G-EG-I** | 3.321 | 45.686 | 42.366 | 12.192 | 110.107 | 10.493 | 2.099 | 31.785 |
| **TOTAL CAR(Mean) \| G-EG-I** | 1.310 | 16.377 | 15.066 | 6.869 | 31.902 | 5.648 | 1.130 | 9.209 |
| **TAC(Mean) \| G-EG-I** | 0.129 | 10.735 | 10.606 | 5.147 | 12.660 | 3.558 | 0.712 | 3.655 |
| **LYCO(Mean) \| G-EG-I** | 0.686 | 10.447 | 9.762 | 3.652 | 10.660 | 3.265 | 0.653 | 3.077 |
| **TPC(Mean) \| G-H/M** | 4755.000 | 39112.500 | 34357.500 | 15963.750 | 91909815.144 | 9586.961 | 2562.223 | 36049995.455 |
| **TOS(Mean) \| G-H/M** | 154.000 | 20010.000 | 19856.000 | 10610.643 | 41766812.901 | 6462.725 | 1727.236 | 16382291.846 |
| **Tanins(Mean) \| G-H/M** | 4450.000 | 12205.000 | 7755.000 | 8930.179 | 4840561.985 | 2200.128 | 588.009 | 1898624.617 |
| **Protease(Mean \| G-H/M** | 7045.000 | 11032.500 | 3987.500 | 8794.107 | 1661057.315 | 1288.820 | 344.452 | 651520.282 |
| **(APX)Mean \| G-H/M** | 460.000 | 1255.000 | 795.000 | 720.179 | 62080.254 | 249.159 | 66.591 | 24349.879 |
| **AsA(Mean) \| G-H/M** | 609.500 | 680.000 | 70.500 | 632.482 | 461.024 | 21.471 | 5.738 | 180.828 |
| **CAT(Mean) \| G-H/M** | 87.500 | 1885.000 | 1797.500 | 415.214 | 199931.220 | 447.137 | 119.502 | 78419.476 |
| **TSP(Mean) \| G-H/M** | 101.050 | 448.063 | 347.013 | 369.400 | 14325.913 | 119.691 | 31.989 | 5619.085 |
| **SOD(Mean) \| G-H/M** | 66.976 | 263.315 | 196.339 | 186.987 | 3270.996 | 57.193 | 15.285 | 1282.990 |
| **MDA(Mean) \| G-H/M** | 145.000 | 222.113 | 77.113 | 179.745 | 717.978 | 26.795 | 7.161 | 281.614 |
| **(TF)Mean \| G-H/M** | 86.594 | 356.103 | 269.508 | 159.389 | 9525.687 | 97.600 | 26.085 | 3736.282 |
| **TOTAL CHLORO(Mean) \| G-H/M** | 40.196 | 266.984 | 226.788 | 136.584 | 5518.548 | 74.287 | 19.854 | 2164.552 |
| **Amylase(Mean) \| G-H/M** | 78.349 | 204.962 | 126.613 | 112.650 | 1224.136 | 34.988 | 9.351 | 480.146 |
| **POD(Mean) \| G-H/M** | 63.500 | 205.150 | 141.650 | 93.557 | 2589.852 | 50.891 | 13.601 | 1015.823 |
| **CHLB(Mean) \| G-H/M** | 13.335 | 192.604 | 179.269 | 73.245 | 2864.310 | 53.519 | 14.304 | 1123.475 |
| **CHLA(Mean) \| G-H/M** | 25.142 | 124.656 | 99.514 | 63.561 | 1282.349 | 35.810 | 9.571 | 502.978 |
| **TS(Mean) \| G-H/M** | 11.657 | 58.492 | 46.835 | 40.328 | 226.373 | 15.046 | 4.021 | 88.791 |
| **NRS(Mean) \| G-H/M** | 4.408 | 44.614 | 40.206 | 29.230 | 190.712 | 13.810 | 3.691 | 74.803 |
| **RS(Mean) \| G-H/M** | 4.661 | 22.080 | 17.419 | 10.606 | 28.741 | 5.361 | 1.433 | 11.273 |
| **TOTAL CAR(Mean) \| G-H/M** | 1.869 | 11.498 | 9.629 | 4.923 | 8.156 | 2.856 | 0.763 | 3.199 |
| **TAC(Mean) \| G-H/M** | 0.423 | 10.563 | 10.139 | 4.377 | 11.000 | 3.317 | 0.886 | 4.314 |
| **LYCO(Mean) \| G-H/M** | 0.816 | 5.023 | 4.207 | 2.695 | 1.827 | 1.352 | 0.361 | 0.717 |
| **TPC(Mean) \| G-AV** | 10760.000 | 42755.000 | 31995.000 | 32428.542 | 65095932.339 | 8068.205 | 2329.090 | 27756998.813 |
| **Protease(Mean \| G-AV** | 7210.000 | 16050.000 | 8840.000 | 12050.833 | 6825314.015 | 2612.530 | 754.173 | 2910323.675 |
| **Tanins(Mean) \| G-AV** | 1825.000 | 13925.000 | 12100.000 | 8003.333 | 11593201.515 | 3404.879 | 982.904 | 4943357.736 |
| **TOS(Mean) \| G-AV** | 310.000 | 12005.000 | 11695.000 | 2116.042 | 10707981.203 | 3272.305 | 944.633 | 4565898.526 |
| **(APX)Mean \| G-AV** | 385.000 | 1280.000 | 895.000 | 786.458 | 92845.975 | 304.706 | 87.961 | 39589.657 |
| **CAT(Mean) \| G-AV** | 85.000 | 1875.000 | 1790.000 | 676.292 | 348867.748 | 590.650 | 170.506 | 148757.708 |
| **AsA(Mean) \| G-AV** | 333.500 | 657.000 | 323.500 | 605.917 | 7584.333 | 87.088 | 25.140 | 3233.971 |
| **POD(Mean) \| G-AV** | 63.500 | 3170.000 | 3106.500 | 505.246 | 736890.464 | 858.423 | 247.805 | 314211.150 |
| **TOTAL CHLORO(Mean) \| G-AV** | 54.444 | 450.310 | 395.865 | 349.793 | 14437.205 | 120.155 | 34.686 | 6156.045 |
| **TSP(Mean) \| G-AV** | 140.792 | 423.295 | 282.503 | 289.981 | 10475.690 | 102.351 | 29.546 | 4466.849 |
| **(TF)Mean \| G-AV** | 132.782 | 358.628 | 225.846 | 289.508 | 3145.644 | 56.086 | 16.191 | 1341.307 |
| **MDA(Mean) \| G-AV** | 132.758 | 300.548 | 167.790 | 224.136 | 1737.532 | 41.684 | 12.033 | 740.886 |
| **CHLB(Mean) \| G-AV** | 19.916 | 274.801 | 254.885 | 197.235 | 5711.665 | 75.576 | 21.817 | 2435.462 |
| **CHLA(Mean) \| G-AV** | 34.653 | 206.295 | 171.642 | 151.055 | 2060.054 | 45.388 | 13.102 | 878.410 |
| **SOD(Mean) \| G-AV** | 78.403 | 262.783 | 184.379 | 141.595 | 2741.545 | 52.360 | 15.115 | 1168.999 |
| **Amylase(Mean) \| G-AV** | 76.594 | 132.642 | 56.047 | 111.609 | 176.236 | 13.275 | 3.832 | 75.147 |
| **TS(Mean) \| G-AV** | 12.349 | 59.981 | 47.632 | 40.504 | 212.838 | 14.589 | 4.211 | 90.754 |
| **NRS(Mean) \| G-AV** | 7.128 | 56.284 | 49.156 | 35.775 | 248.776 | 15.773 | 4.553 | 106.078 |
| **TOTAL CAR(Mean) \| G-AV** | 2.411 | 17.872 | 15.461 | 13.738 | 19.153 | 4.376 | 1.263 | 8.167 |
| **LYCO(Mean) \| G-AV** | 1.077 | 10.042 | 8.965 | 7.572 | 6.776 | 2.603 | 0.751 | 2.889 |
| **TAC(Mean) \| G-AV** | 0.529 | 12.667 | 12.138 | 5.735 | 18.939 | 4.352 | 1.256 | 8.076 |
| **RS(Mean) \| G-AV** | 2.883 | 14.852 | 11.968 | 5.259 | 10.035 | 3.168 | 0.914 | 4.279 |
| **TPC(Mean) \| G-OG** | 25955.000 | 46860.000 | 20905.000 | 32221.250 | 96237260.417 | 9810.059 | 4905.030 | 78577394.088 |
| **Protease(Mean \| G-OG** | 10202.500 | 14190.000 | 3987.500 | 12031.875 | 3452889.063 | 1858.195 | 929.098 | 2819272.114 |
| **Tanins(Mean) \| G-OG** | 5600.000 | 11260.000 | 5660.000 | 9136.875 | 7300522.396 | 2701.948 | 1350.974 | 5960851.575 |
| **(APX)Mean \| G-OG** | 775.000 | 1315.000 | 540.000 | 974.375 | 61426.563 | 247.844 | 123.922 | 50154.578 |
| **AsA(Mean) \| G-OG** | 603.750 | 628.000 | 24.250 | 617.500 | 135.375 | 11.635 | 5.818 | 110.533 |
| **CAT(Mean) \| G-OG** | 229.000 | 1275.000 | 1046.000 | 598.500 | 219785.667 | 468.813 | 234.407 | 179454.245 |
| **TOS(Mean) \| G-OG** | 97.500 | 462.500 | 365.000 | 345.250 | 29581.083 | 171.992 | 85.996 | 24152.853 |
| **TOTAL CHLORO(Mean) \| G-OG** | 284.390 | 366.082 | 81.692 | 329.037 | 1138.994 | 33.749 | 16.874 | 929.985 |
| **(TF)Mean \| G-OG** | 277.472 | 317.125 | 39.654 | 302.691 | 312.787 | 17.686 | 8.843 | 255.389 |
| **TSP(Mean) \| G-OG** | 64.827 | 385.000 | 320.173 | 239.207 | 19600.168 | 140.001 | 70.000 | 16003.470 |
| **MDA(Mean) \| G-OG** | 190.823 | 242.419 | 51.597 | 210.996 | 510.672 | 22.598 | 11.299 | 416.962 |
| **CHLB(Mean) \| G-OG** | 165.490 | 202.951 | 37.461 | 184.361 | 243.163 | 15.594 | 7.797 | 198.541 |
| **CHLA(Mean) \| G-OG** | 131.221 | 163.432 | 32.211 | 151.154 | 222.556 | 14.918 | 7.459 | 181.716 |
| **POD(Mean) \| G-OG** | 63.700 | 191.600 | 127.900 | 130.313 | 2758.864 | 52.525 | 26.262 | 2252.603 |
| **Amylase(Mean) \| G-OG** | 113.896 | 118.349 | 4.453 | 116.153 | 3.858 | 1.964 | 0.982 | 3.150 |
| **SOD(Mean) \| G-OG** | 91.174 | 146.457 | 55.283 | 116.033 | 548.706 | 23.424 | 11.712 | 448.016 |
| **TS(Mean) \| G-OG** | 31.217 | 46.519 | 15.302 | 40.178 | 41.316 | 6.428 | 3.214 | 33.734 |
| **NRS(Mean) \| G-OG** | 29.625 | 44.718 | 15.093 | 37.249 | 38.858 | 6.234 | 3.117 | 31.728 |
| **TOTAL CAR(Mean) \| G-OG** | 12.716 | 14.884 | 2.168 | 13.506 | 1.023 | 1.011 | 0.506 | 0.835 |
| **LYCO(Mean) \| G-OG** | 7.263 | 7.918 | 0.655 | 7.503 | 0.082 | 0.286 | 0.143 | 0.067 |
| **TAC(Mean) \| G-OG** | 0.494 | 12.839 | 12.345 | 6.049 | 27.586 | 5.252 | 2.626 | 22.524 |
| **RS(Mean) \| G-OG** | 3.192 | 7.576 | 4.384 | 5.229 | 3.236 | 1.799 | 0.899 | 2.642 |
